# Polyamines and Legumes: Joint Stories of Stress, Nitrogen Fixation and Environment

**DOI:** 10.3389/fpls.2019.01415

**Published:** 2019-11-04

**Authors:** Ana Bernardina Menéndez, Pablo Ignacio Calzadilla, Pedro Alfonso Sansberro, Fabiana Daniela Espasandin, Ayelén Gazquez, César Daniel Bordenave, Santiago Javier Maiale, Andrés Alberto Rodríguez, Vanina Giselle Maguire, Maria Paula Campestre, Andrés Garriz, Franco Rubén Rossi, Fernando Matias Romero, Leandro Solmi, Maria Soraya Salloum, Mariela Inés Monteoliva, Julio Humberto Debat, Oscar Adolfo Ruiz

**Affiliations:** ^1^Instituto Tecnológico de Chascomús (INTECH), UNSAM-CONICET, Chascomús, Argentina; ^2^Departamento de Biodiversidad y Biología Experimental, Facultad de Ciencias Exactas y Naturales, UBA-CONICET, Buenos Aires, Argentina; ^3^Instituto de Botánica del Nordeste (IBONE), UNNE-CONICET, Corrientes, Argentina; ^4^Instituto de Fisiología y Recursos Genéticos Vegetales (IFRGV) Ing “Victorio S Trippi,” Instituto Nacional de Tecnología Agropecuaria (INTA), Córdoba, Argentina; ^5^Instituto de Patología Vegetal (IPAVE) Ing “Sergio Nome,” Instituto Nacional de Tecnología Agropecuaria (INTA), Córdoba, Argentina

**Keywords:** legume, plant polyamines, plant stress and adaptation, symbionts, constrained environments

## Abstract

Polyamines (PAs) are natural aliphatic amines involved in many physiological processes in almost all living organisms, including responses to abiotic stresses and microbial interactions. On other hand, the family *Leguminosae* constitutes an economically and ecologically key botanical group for humans, being also regarded as the most important protein source for livestock. This review presents the profuse evidence that relates changes in PAs levels during responses to biotic and abiotic stresses in model and cultivable species within *Leguminosae* and examines the unreviewed information regarding their potential roles in the functioning of symbiotic interactions with nitrogen-fixing bacteria and arbuscular mycorrhizae in this family. As linking plant physiological behavior with “big data” available in “omics” is an essential step to improve our understanding of legumes responses to global change, we also examined integrative MultiOmics approaches available to decrypt the interface legumes-PAs-abiotic and biotic stress interactions. These approaches are expected to accelerate the identification of stress tolerant phenotypes and the design of new biotechnological strategies to increase their yield and adaptation to marginal environments, making better use of available plant genetic resources.

## Introduction

**Figure 1:**
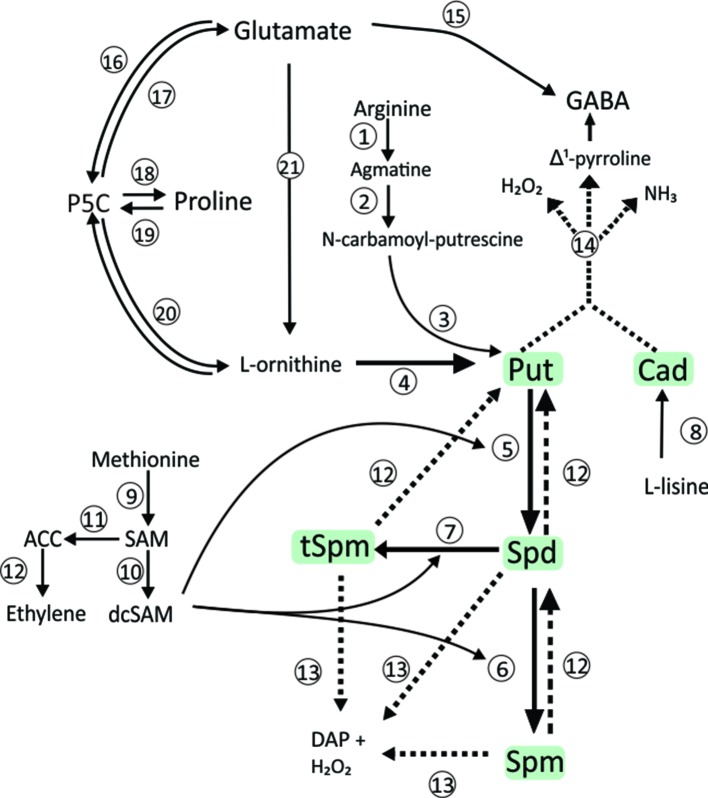
Polyamine metabolism. Biosynthetic pathways for PAs and related metabolites are indicated by continuous lines. Dashed lines show the metabolites from the catabolism of the Pas. Abbreviations: Put, putrescine; Cad, Cadaverine; Spd, spermidine; Spm, spermine; tSpm, thermospermine; SAM, S-adenosylmethionine; dcSAM, decarboxylated S-adenosylmethionine; ACC, aminocyclopropane carboxylic acid; P5C, glutamate-5-semialdehyde. Numbers refer to enzymes: 1, arginine decarboxylase (ADC, EC 4.1.1.19); 2, agmatine iminohydrolase (EC 3.5.3.12); 3, N-carbamoylputrescine amido-hydrolase (EC 3.5.1.53); 4, ornithine decarboxylase (ODC, EC 4.1.1.17); 5, spermidine synthase (SPDS, EC 2.5.1.16); 6, spermine synthase (SPMS, EC 2.5.1.22); 7, thermospermine synthase; 8, L-lysine decarboxylase (EC 4.1.1.18); 9, SAM synthetase; 10, SAM decarboxylase (SAMDC, EC 4.1.1.50); 11, ACC synthase (EC 4.4.1.14); 12, ACC oxidase (EC 1.14.17.4); 12, back-conversion polyamine oxidase (non-specific polyamine oxidase, EC 1.5.3.17); 13, terminal catabolism polyamine oxidase (propane-1,3-diamine-forming, EC 1.5.3.14); 14, diamine oxidase (DAO, EC 1.4.3.6); 15, GAD (glutamate decarboxylase, EC 4.1.1.15); 16, P5CDH (Δ1-pyrroline-5-carboxylate dehydrogenase, EC 1.5.1.12); 17,P5CS (L-glutamate γ-semialdehyde dehydrogenase, EC 1.2.1.88); 18, P5CR (Δ1-pyrroline-5-carboxylate reductase, EC 1.5.1.2); 19, ProDH (proline dehydrogenase, EC 1.5.99.8); 20, OAT (ornithine δ-aminotransferase, EC 2.6.1.13); 21, Glutamate to Ornithine subpathway (five steps subpathway catalyzed by EC 2.3.1.1, EC 2.7.2.8, EC 1.2.1.38, EC 2.6.1.11 and EC 3.5.1.16).

Polyamines (PAs) are organic polycations, acknowledged as regulators of plant growth, development and stress responses, being putrescine (Put), spermidine (Spd), and spermine (Spm) the most related to this physiological role (Cohen, 1998). A high number of metabolites and enzymes participate in PAs metabolism (see Calzadilla et al., 2014 for an extensive description). The diamine Put can be synthesized directly from ornithine by the enzyme ornithine decarboxylase (ODC, EC 4.1.1.17) or indirectly, *via* a series of intermediates following decarboxylation of arginine by arginine decarboxylase (ADC, EC 4.1.1.19) (**Figure 1**). In turn, Spd and Spm are synthesized from Put by successive additions of aminopropyl groups provided by decarboxylated S-adenosylmethionine (SAM), a metabolite derived from the S-adenosylmethionine decarboxylase (SAMDC, EC 4.1.1.50) activity. The aminopropyl additions to Put are catalyzed by the aminopropyl-transferases Spd (EC 2.5.1.16) and Spm synthases (EC 2.5.1.22). Both ADC and ODC pathways occur in higher plants and bacteria (**Figure 1**).

PAs are present in cells as free and bound forms, in variable amounts, depending on the species and developmental stage (Jiménez-Bremont et al., 2007; Alcázar et al., 2010;Hussain et al., 2011 ). The free forms of PAs show water-soluble properties and therefore, are readily translocated within cells. They may cause conformational stabilization/destabilization of DNA, RNA, chromatin, and proteins due to their ability to form electrostatic linkages with negatively charged molecules (Alcázar et al., 2010; Wimalasekera et al., 2011). PAs can stabilize membranes or nucleic acids, by binding to their negative surfaces (Galston and Sawhney, 1990; Kusano et al., 2008). Although the H_2_O_2_ derived from PAs catabolism contributes to reactive oxygen species (ROS) (Gonzalez et al., 2011), PAs can also act as ROS scavengers and activate antioxidant enzymes (Pottosin et al., 2014a). In addition, PAs display effects on vacuolar channels and cation transport in plants (Pottosin and Muñiz, 2002; Pottosin and Shabala, 2014; Zepeda-Jazo and Pottosin, 2018). Notwithstanding the precise molecular mechanisms by which PAs control plant responses to abiotic stress remain unknown, several aspects about their apparently clashing roles in the development have been reviewed the last years (e.g., Minocha et al., 2014; Singh et al., 2018; Chen et al., 2019a). These aspects include the involvement of PAs signaling in direct interactions with different metabolic routes like intricate hormonal cross-talks, the nitric oxide formation and the modulation of ion channel activities, and Ca^2+^ homeostasis (Tun et al., 2006; Yamasaki and Cohen, 2006;Alcázar et al., 2010; Bitrián et al., 2012; Minocha et al., 2014; Singh et al., 2018; Podlešáková et al., 2019).

The family *Leguminosae*, containing close to 770 genera and over 19,500 species (LPWG, 2017), is the third largest Angiosperms family in terms of species numbers after *Asteraceae* and *Orchidaceae*. A considerable number of features make legumes an excellent model system to study the different aspects of PAs metabolism. Legumes are critical components of natural and agricultural ecosystems (Escaray et al., 2012). Indeed, some legumes such as soybean (*Glycine max* L. Merr.) and peanut (*Arachis hypogaea*) are food crops of primary economic importance for livestock and human consumption, as their seeds are rich in proteins, carbohydrates, and oils (Duranti and Gius, 1997; Graham and Vance, 2003). In addition, legumes are characterized by their ability to establish symbiotic interactions with nitrogen fixation bacteria (NFB) and arbuscular mycorrhizal fungi (AMF), which help at plant nutrition and adaptation to soils offering different environmental constraints (Escaray et al., 2012; Gibson et al., 2008).

The ability of legumes to establish symbiotic interactions with nitrogen-fixing bacteria and arbuscular mycorrhizae fungi gives to some legume species that can exploit their molecular machinery “pioneer” attributes, with better competition in nutrient-poor soils and higher adaptation to restricted environments. However, many legume crops may be affected by several biotic and abiotic stresses, whereby maintaining their yields safe from adverse environmental conditions is probably one of the biggest challenges facing modern agriculture. Therefore, the obtaining of vigorous genotypes with higher tolerance to abiotic and biotic stresses has turned an increasingly important biotechnological target. At this scenario, PAs can play an important role, and genetic manipulation of crop plants with genes encoding PAs biosynthetic pathway enzymes is envisioned as a strategy to achieve plants with improved stress tolerance and symbiotic performance.

In order to support legume crops yields and to understand their limitations, biological, physiological, and diverse omics studies have been carried out in the last 80 years. More recently, genomic tools (genome sequences, expressed sequence tags, oligonucleotide, and cDNA microarrays) have emerged, along with comprehensive databases such as the Legume Information System (http://www.comparative-legumes.org). In particular, genomic sequencing of *Medicago truncatula* (Young et al., 2011) and *Lotus japonicus* (Sato et al., 2008), differing in their patterns of root nodule formation (Barker et al., 1990; Handberg and Stougaard, 1992), and also of soybean (Schmutz et al., 2010), pigeon pea (Varshney et al., 2012), and chickpea (Varshney et al., 2013) have provided a solid framework to explore PAs metabolism for legume crop improvement.

The recent developments in legumes research are fundamental to sustain food security at a global level. However, the current paradigm in plant science is characterized by a disconnection of ecophysiology and “omics,” which have been established in parallel, with only exceptional cross-talks for the past 20 years. A new field has been proposed in order to capitalize the concurrent advances of both areas into a single discipline: “ecophysiolomics” (Flexas and Gago, 2018). This multidisciplinary approach would require joining forces, equipment, and abilities in the context of user-friendly integrative bioinformatics resources and concurrent shared research protocols. This utopic collaborative environment is essential to advance in the understanding of legumes at levels ranging from cellular to agroecosystem scales. Then breeders may be able to use this understanding and translate it into practices or biotechnological tools.

This review explores the contribution made by studies on legume species on the basic knowledge of PAs metabolism, their role in tolerance to biotic and abiotic stresses, and the establishment of mutualistic relationships relevant to the physiology of plants and the environment. As significant future challenges are the development and implementation of progressive methods of genetic improvement oriented to develop varieties of legumes that have genetic recovery capacity against environmental stresses, this review will also get a glimpse of the state-of-the-art toolkit landscape in legume research.

### Polyamines and Abiotic Stress in Legumes

#### Drought Stress

Exogenous PAs application has shown to mitigate drought stress in several legumes such as *Phaseolus vulgaris* (Torabian et al., 2018a; Torabian et al., 2018b), *Trifolium repens* (Zhang et al., 2018), and *Vigna radiata* (Farhangi-Abriz et al., 2017), whereas the mechanisms involved in the PAs-mediated alleviation of drought include the crosstalk with several phytohormones, the improvement of plant water status, stress signaling, antioxidant biosynthesis, melatonin production, and DNA protection.

Plant acclimation strategies to water deficit are based on short-term osmotic responses (Tardieu et al., 2018), and abscisic acid (ABA) and other phytohormones are acknowledged by their involvement in fast tolerance response to dehydration (Ullah et al., 2018). Several studies carried out with ABA-deficient mutants of non-legume species confirmed that ABA mediates the drought upregulation of *ADC2*, *SPDS1*, and *SPMS* genes at the transcriptional level (Alcázar et al., 2010). Legume-base studies have also provided significant pieces of evidence pointing to PAs playing a role in the regulation of short-term osmotic responses by interacting with phytohormones. For example, Espasandin et al. (2014) showed that the overexpression of the oat *ADC2* gene in *Lotus tenuis* plants, using a heterologous oat *ADC* gene under the control of a drought/ABA-inducible promoter *RD29A*, increased Put content in shoots of drought-stressed plants. These authors revealed that Put controls ABA biosynthesis in response to drought by modulating the expression of 9-cis-epoxycarotenoid dioxygenase (NCED). Drought increased the expression of oat ADC, total ADC activity, and Put content with only minor variations in Spd and Spm. The wild-type plants showed relatively smaller changes in PAs metabolism upon exposure to water shortage. Concomitantly, the higher Put content in transgenic lines significantly increased the NCED expression, suggesting the possibility of transcriptional regulation of ABA synthesis by Put. All these results underpin the theory that Put and ABA are assimilated in a positive feedback loop in response to osmotic stress (Singh et al., 2018). In contrast, Spd application had no significant effect on ABA accumulation in *T. repens*, although it led to increased GA and cytokinin (CTK) content as well as decreased indole-3-acetic acid (IAA) content under water deficit condition (Li et al., 2016a). In this species, IAA-PAs crosstalk was involved in the improvement of antioxidant defense and osmotic adjustment conferring plant tolerance to water stress (Li et al., 2018a). Moreover, crosstalk with ABA, IAA, and CKs is predictable since these phytohormones modulate root architecture to maintain the water homeostasis to cope with the long-term water shortage (Khan et al., 2016). In effect, in drought-stressed *L. tenuis* (Espasandin et al., 2014), *Cicer arietinum, G. max* (Nayyar et al., 2005), and *Medicago sativa* (Zeid and Shedeed, 2006), the rise in Put level either by over-expression of genes related to PAs biosynthesis, or exogenous application promoted root development. These effects may also be related to the involvement of PAs in the control of cell division and differentiation, which plays an essential role in the root apex and during lateral root formation (Couée et al., 2004). Further proteomic analysis in *T. repens* (Li et al., 2018b) revealed that PAs could be one key adaptive response against drought stress, through the regulation of growth, ribosome, amino acid and energy metabolism, and antioxidant reactions.

Drought induces oxidative stress due to an imbalance between ROS formation and scavenging (Fariduddin et al., 2013). The excessive ROS production, in turn, decreases the membrane fluidity and damages membrane proteins inactivating the related receptors, enzymes, and ion channels (Gill and Tuteja, 2010). PAs, mainly Put and Spm, are responsible for the scavenging of ROS and can indirectly affect the activities of the involved enzymes including catalase, peroxidases, and superoxide dismutase (Alcázar et al., 2010; Minocha et al., 2014; Sánchez-Rodríguez et al., 2016). A body of evidence obtained from experiments using water-stressed legumes has also contributed to support that PAs could act as a signal molecule or as antioxidants during the stress response. PAs treatment increases the activities of antioxidant enzymes and reduces the oxidative damages in *C. arietinum* (Nayyar and Chander, 2004). Likewise, the exogenous application of Spm and Spd regulates antioxidant defense system by increasing the reduced glutathione concentration or catalase activity in drought-stressed *G. max* (Radhakrishnan and Lee, 2013) and *T. repens* (Li et al., 2014a). Furthermore, PAs synthesis and oxidation have shown to improve H_2_O_2_-induced antioxidant protection in *M. sativa* (Guo et al., 2014).

Chloroplasts employ several strategies to cope with energy imbalances and prevent ROS formation, which was recently reviewed by Vanlerberghe et al. (2016). In such circumstances, Spm plays an essential role in protecting the photosynthetic apparatus by interaction with photosystem II and light harvesting complex (LCH) proteins, preserving the integrity of the thylakoid membranes structure (Hamdani et al., 2011), helping in the maintenance of the photosynthetic activity. Drought-sensitive *P. vulgaris* plants presented significant decreases in the contents of all PAs associated with thylakoids isolated from plants growing in sorbitol and salt conditions (Legocka and Sobieszczuk-Nowicka, 2012), suggesting that thylakoid-associated PAs would be good markers of plant stress tolerance. Also, Spm application to *G. max* leaves reduced osmotic stress-induced losses in chlorophyll, carotenoid, and protein levels (Radhakrishnan and Lee, 2013).

It is known that PAs actively participate in stress signaling through an intricate crosstalk with several signal molecules (Marco et al., 2011; Shi and Chan, 2014). Nitric oxide (NO) is a key signaling molecule that can also be induced by PAs. In *T. repens*, Spd played a role in drought stress-activated pathways associated with NO release, which mediated antioxidant defense, contributing to drought tolerance in this plant (Peng et al., 2016), whereas in *Vicia faba*, NO accumulation proved necessary for ABA-induced closure of stomata (Garcia-Mata and Lamattina, 2002). On other hand, hydrogen sulfide (H_2_S) is currently regarded as a novel gaseous signaling molecule in plants during environmental stress response (Li et al., 2019). In *T. repens*, dehydration or exogenous application of Spd caused a quick H_2_S accumulation, followed by significant improvement of antioxidant activities and increased transcript levels of several transcription factors and genes encoding antioxidant enzymes, all associated with dehydration tolerance. Further analyses using NO and H_2_S scavengers led to the notion that Spd-induced H_2_O_2_ could be an upstream signal molecule of NO and H_2_S, whereas Spd-induced H_2_S might act as the downstream signaling of NO in *T. repens* leaves.

Drought perturbs photosynthesis due to CO_2_ limitations resulting from stomatal closure, and biochemical restrictions associated with the accumulation of reducing power (Pinheiro and Chaves, 2010). PAs are shown to regulate ion channel and Ca^2+^ homeostasis, and it is known that changes of free Ca^2+^ in the cytoplasm of guard cells are responsible for stomatal movement (Allen and Sanders, 1996; Peiter et al., 2005). Several works performed on legumes have also suggested that the interaction between PA-induced H_2_O_2_ and Ca^2+^ signaling plays a role in stomata movement. In *V. faba*, diamine oxidase (DAO) catalyses the degradation of Put to produce H_2_O_2_, thereby elevating the Ca^2+^ level in guard cells (An et al., 2008). PAs-regulated tolerance to water stress in *T. repens* was associated with antioxidant defenses and dehydrins *via* their involvement in the Ca^2+^ messenger system and H_2_O_2_ signaling pathways (Li et al., 2015a). The constitutive overexpression of oat arginine decarboxylase 2 (*ADC2*) gene increases the net CO_2_ assimilation rate in *M. truncatula* stressed leaves at the expenses of an increase in the stomatal conductance and transpiration (Duque et al., 2016). Contrarily, the *ADC2* overexpression driven by the stress-inducible *RD29A* promoter improved drought tolerance in *L. tenuis* plants subjected to a gradual decrease in water availability, by inducing stomatal closure to reduce transpiration (Espasandin et al., 2014). As the intensity of stress increases, PAs promote osmoregulation and preserves the leaf relative water content by inducing the accumulation of proline, an amino acid playing a highly beneficial role in plants exposed to osmotic stress conditions (Hayat et al., 2012; in *C. arietinum, G. max*, and *L. tenuis*). Furthermore, pre-treatment of *T. repens* seeds by soaking with Spd 30 µM for 90 min increased α- and β-amylase activities accelerating the starch metabolism during germination under low soil water content (Li et al., 2014b). Likewise, pre-treatment of 7-day-old *T. repens* plants with Spd 500 µM for 7 days speed up the water-soluble sugar, sucrose, fructose, sorbitol, and dehydrins accumulation in drought-stressed leaves (Li et al., 2015b). Also, γ-aminobutyric acid (GABA), a product of Put catabolism by DAO and terminal catabolism of Spd (Xing et al., 2007) may act as an osmolyte to reduce the loss of cellular water and also protects the plant from stress by regulating cell pH (Podlešáková et al., 2019). In fact, treatment with exogenous GABA led to improved drought tolerance of *T. repens*, associated with a positive regulation in the GABA-shunt and PAs metabolism (Yong et al., 2017).

Legumes as model species have facilitated the study of unique biological mechanisms used by plants in response to stress. *M. truncatula* has been valuable to reveal the role of *MtSPDS* and *MtSPMS* genes encoding Spd synthase and Spm/Spd synthase, respectively, as part of the molecular mechanisms underlying DNA damage response in legumes (Pagano et al., 2019). In *M. sativa*, melatonin pre-treatment exerted, through PAs modulation, a protective effect on plants against waterlogging (Zhang et al., 2014). Interestingly, melatonin improved tolerance to salt and drought stresses in *G. max* and *V. faba* as well (Wei et al., 2014; Dawood and El-Awadi, 2015). It would be worthwhile to test whether melatonin also induces the development of lateral root primordia through the stimulation of polyamine oxydase (*PAO1*) expression as it was recently shown for *Solanum lycopersicum* (Chen et al., 2019b). Finally, physiological and iTRAQ-based proteomic analyses on Spd-treated *T. repens* explained Spd-induced physiological effects associated with improved drought tolerance through the higher abundance of differential expressed proteins involved in protein (ribosomal and chaperone proteins) and amino acids biosynthesis, in carbon and energy metabolisms, in antioxidants (ascorbate peroxidase, glutathione peroxidase, and dehydrins), and in GA and ABA signaling pathways (Li et al., 2016b).

#### Salt Stress

Salinity is a severe problem for crops worldwide (Flowers, 2004), affecting around 800 million ha. (FAO, 2008). Salt stress disturbs plants in a two-phases way; the first involves the reduction of shoot growth due to the osmotic stress caused by high salt concentration in the rhizosphere, and the second is driven by the accumulation of toxic ions (Munns and Tester, 2008). Supplementation of salt affected plants with exogenous PAs led to improved toxicity symptoms and plant growth in several legume species such as *V. radiata* (Nahar et al., 2016a) and *G. max* (Zhang et al., 2014).

Experiments using *L. tenuis* (formerly *Lotus glaber*) as a model to test the hypothesis that free Spd and Spm are biochemical indicators of the salt stress response, have shown that salt induced a decrease and an increase of free Spd and Spm, respectively (Sanchez et al., 2005). These results suggest the lack of a relationship between the salt induced reduction of growth rate and Spd content, while Spm might be related to stress signaling. Several studies using “omics” techniques have revealed that salinity modulates the expression of genes involved in PAs metabolism. In *C. arietinum* (chickpea) roots, it was shown that salt induced the up-regulation of *ADC* and *SAMDC* (Molina, 2008).Sánchez et al. (2011) analyzed the contrasting responses to salinity of six *Lotus* species by using comparative ionomics, transcriptomics, and metabolomics. These authors found that many salt-elicited genes of PAs metabolism showed a similar gene-expression profile in sensitive and tolerant species. These shared transcripts included many genes previously implicated in plant stress such as enzymes of PAs biosynthesis and catabolism, proline oxidase, polyamine oxidase, SAMDC, and Spm synthase. Also, recent metabolomic studies revealed that salinity increases the content of different free PAs in legumes, in *Prosopis strombulifera* leaves (Llanes et al., 2016) and *G. max* roots (Jiao et al., 2018). Interestingly, *Pr. strombulifera* showed an increase of cadaverine (Cad), an uncommon diamine that characterizes the legume family (Jancewicz et al., 2016).

The fact that salinity induces osmotic stress and redox imbalances implies that some stress symptoms and mechanisms for their mitigation are shared with drought, including improvement of plant water status, stress signaling, and synthesis of antioxidants (see previous section). In this regard, several studies on legume species have contributed to reveal possible roles of PAs on key physiological responses of plants to salt stress. In NaCl-treated *Pr. strombulifera*, Put accumulation was related to the antioxidant defense system in this species (Reginato et al., 2012). In *V. radiata*, supplementation with exogenous Put resulted in better seedling growth, associated with enhanced glutathione and ascorbate contents, increased activities of antioxidant enzymes and glyoxalase enzyme, and reduced cellular Na^+^ (Nahar et al., 2016a). GABA is also an important intermediate involved in ROS scavenging under abiotic stress and has been proposed that it contributes to stress protection (Bouche and Fromm, 2004; Liu et al., 2011). NaCl (100 mM) stress induced higher GABA accumulation in *G. max* through DAO activity stimulation, whereas GABA levels were reduced concomitantly to PAs increment during stress recovery (Xing et al., 2007). A regulatory role of GABA on PAs genes the expression of emerged from salt-treated plants of the shrub *Caragana intermedia* (Shi et al., 2010), and *V. faba* plants grown under hypoxia (Yang et al., 2013; Yang et al., 2018). Using quantitative profiling, Dias et al. (2015) detected a decrease of Put level in tolerant and sensitive chickpea genotypes subjected to stress, whereas the sensitive genotype also had reduced GABA. These results were confirmed by a transcriptomic analysis (Liu et al., 2019), indicating that the expression of key genes of the GABA shunt pathway and polyamine degradation was positively induced in soybean leaves under saline stress. Interestingly, Ca regulated GABA metabolism pathways in germinating soybean under NaCl stress, by changing the contribution ratio of GABA shunt and polyamine degradation pathway for GABA formation (Yin et al., 2014). Taken together, these results strongly support the idea that sustaining GABA levels could be a major strategy to cope with salinity in legumes.

It is well-known that both PAs and the osmolyte proline possess a common precursor: glutamate. Glutamate can be directly converted to proline through the glutamate Δ^1^-pyrroline-5-carboxylate pathway or indirectly to PAs *via* its acetylation in ornithine and arginine (Hayat et al., 2012). This connection between PAs and proline metabolisms would imply that considerable stress-induced changes in the pool of PAs could cause a shift in the proline synthesis pathway. Effectively, in 2-week-old soybean seedlings subjected to NaCl, Su and Bai (2008) found a negative correlation between proline accumulation and endogenous Put content. However, in long-term salt-stressed *L. glaber*, PAs and proline accumulations were not correlated (Sanchez et al., 2005). Such a divergence is not surprising, since it was recently found in wheat that production of proline was partly regulated independently, and not in an antagonistic manner from the PAs synthesis (Pál et al., 2018).

Other works have addressed the salt-specific, ionic homeostasis response, through the regulation of non-selective cation channels. These protein channels are known to be PAs targets (Liu et al., 2000) and their blockage by PAs led to the prevention of the salt-induced K^+^ efflux from *Pisum sativum* mesophyll cells (Shabala et al., 2007). In the last species, PAs were also shown to interact with ROS to alter intracellular Ca^2+^ homeostasis by modulating both Ca^2+^ influx and efflux transport systems at the root cell plasma membrane (Zepeda-Jazo et al., 2011). Pottosin et al. (2012) found a synergism between PAs and ROS in the induction of passive Ca^2+^ and K^+^ fluxes in roots, which would impact K^+^ homeostasis and Ca^2+^ signaling under stress. Also, in *P. sativum* roots, PAs caused plasma membrane depolarization, activated Ca^2+^, and modulated H^+^-ATPase pump activity (Pottosin et al., 2014b). Taken together, this data suggests a possible link between PAs and Ca^2+^ homeostasis, and stress responses in legumes, which deserves further attention.

Most of the studies mentioned above have focused on the effects caused by salt after many hours or days of stress treatment. Geilfus et al. (2015) performed an extensive metabolomic analysis of the fast responses to moderate NaCl stress in *V. faba*. The metabolite profile revealed a rapid reduction in the content of leaf Spd, a PA that is especially relevant for H_2_O_2_ production during its catabolism by PAO. This fast reduction of leaf Spd was suggested to contribute to the excessive ROS production observed in these plants, which started simultaneously 45 min after NaCl treatment. However, authors did not report whether that early oxidative burst served as a beneficial event under NaCl stress or caused oxidative damage. A hint that PAs catabolism would be beneficial for plant growth was provided by Campestre et al. (2011). These authors treated 7 day-old *G. max* seedlings with NaCl demonstrated that ROS generated as a consequence of PAs catabolism participate in the hypocotyl elongation of stressed plants, as apoplastic ROS promote the leaf elongation under salinity (Rodríguez et al., 2009).

A significant number of additional works have analyzed the salt-induced changes in the PAs profiles of different legumes like *G. max* (Zhang et al., 2014), *P. vulgaris* (Zapata et al., 2008;Shevyakova et al., 2013; López-Gómez et al., 2014, Talaat, 2015; López-Gómez et al., 2016) and *L. tenuis* (Maiale et al., 2004; Sanchez et al., 2005; Sannazzaro et al., 2007). Taken together, these studies performed on legumes indicate that PAs metabolism is involved in many physiological processes affected by salinity, through specific mechanisms which contribute to counteract the effects of salinity.

#### Heavy Metals and Extreme Environments

Heavy metals-derived soil pollution is one of the most serious worldwide environmental problems (Ruttens et al., 2006), which poses a health risk to humans and animals through the food chain or contaminated drinking water (Granero and Domingo, 2002). In plants, heavy metal toxicity may cause chlorosis, necrosis and several alterations in plant phenotype (Benzarti et al., 2008). One of the symptoms of metal phytotoxicity is oxidative stress, so plant defense system includes a battery of diverse antioxidants (Hossain et al., 2012). Although Lin and Kao, (1999) stated almost 20 years ago that Put accumulation may be part of the syndrome of copper toxicity, more recent works indicated that PAs biosynthesis in the presence of heavy metals such as Zn, Cu, Cd, Mn, Pb, Fe, and Al could be exerting an antioxidant function by protecting the tissues from the metals-induced oxidative damage, although the precise mechanism of protection still needs to be elucidated (Wolff et al., 1995; Franchin et al., 2007; Groppa et al., 2007).

Heavy metal stress induced the accumulation of Spd in *Cajanus cajan* (Radadiya et al., 2016) and of Put, Spm, and Spd in *V. radiata* (Choudhary and Singh, 2000). In *G. max*, the involvement of PAs seedlings response to cadmium stress was revealed by the induction of *SAMDC* after 3 and 24 h of Cd treatment (Chmielowska-Bąk et al., 2013). Also, in *V. radiata*, exogenous Spm application reduced content, accumulation, and translocation of Cd to different plant organs, which consequently reduced ROS production and oxidative damage, thus preventing chlorophyll degradation (Nahar et al., 2016b).

Increased solubilized Al may result from soil acidification (common in tropical and subtropical regions; Hoekenga et al., 2003; Rahman et al., 2018), limiting crop production (Kochian et al., 2004). Aluminum toxicity induces oxidative damage by overproducing reactive oxygen species (ROS; H_2_O_2_ and O_2_^•−^). Exogenous Spd induced the protection of photosynthetic pigment and improved growth performances of *V. radiata* against Al stress, by regulating proline, and activating enzymatic and non-enzymatic antioxidant defenses (Nahar et al., 2017). Favoring NO over H_2_O_2_ production by the application of ascorbate (a H_2_O_2_ scavenger), a higher expression of the *ADC* gene and increased PAs biosynthesis and GABA were associated with improved Pb tolerance in *Prosopis farcta* (Zafari et al, 2017).

On other hand, evolution has allowed plants to adapt to extreme environments, including severe cold, high salinity, drought conditions, intense heat, acid soils, and desert environments (Oh et al., 2013). Plants that inhabit those environments and can grow optimally at or near those extreme ranges are usually called extremophiles and harbor a range of mechanisms that help them to withstand these extreme conditions. The literature addressing PAs metabolism in legumes using species that naturally occur in these environments is null, but some information is available from studies performed on common legume crops cultivated under heat conditions and high heavy metal levels. Heat stress induced accumulation of PAs in heat-tolerant *A. hypogaea*, a phenomenon already observed on cell cultures of heat tolerant plants and absent on susceptible ones (Königshofer and Lechner, 2002; Raval et al., 2018). A protective role of PAs in this condition has also been demonstrated in *V. radiata* and *C. cajan* under heat stress. Application of either 0.1 or 1 mM of exogenous Put, Spd, or Spm in *V. radiata* seedlings resulted in an enhancement of the thermal protection, as shown by growth parameters (Basra et al., 1997). Also, exogenous Put or Spm application (0.5 mM) to *C. cajan* seeds resulted in a higher germination percentage, and when applied on the seedlings, it resulted in the accumulation of proline (Silva et al., 2015). The protective role of the *de novo* PAs synthesis was revealed by treating *V. radiata* seedlings with inhibitors of PAs biosynthesis [2 mM and 4 mM of either difluoromethylornithine (DFMO) or DFMA], which rendered them vulnerable to heat-shock (lower root, hypocotyl and whole seedling length), being the inhibitory effect reversed by exogenous Put (1 mM; Basra et al., 1997). Overall, PAs accumulation seems to protect legumes against extreme environments and heavy metal toxicity. Reports suggest that the protective effect would be mainly through modulation of the redox metabolism and osmo-protection. However, further research is needed in order to unravel the connection between PAs pathways and the observed mitigation effects.

### Polyamines and Biotic Interactions in Legumes

#### Plant Pathogens and Herbivores

Accumulating evidence indicates that PAs play an essential role in maintaining cell viability during biotic stress, but they also participate in the elicitation of plant defense responses, either functioning as signaling molecules or rather enabling the generation of ROS through their oxidation by PAO. Part of the information supporting the important role played by PAs oxidation during plant response to microbes (reviewed by Jiménez Bremont et al., 2014) has emerged from studies using legume species. These studies suggest that Put oxidation might play a critical role in plant defense against fungal pathogens.

PAs metabolism is tightly regulated during plant-pathogenic interactions (Jiménez Bremont et al., 2014, Rossi et al., 2018). Transcriptomic data indicate that the regulation of PAs metabolism occurring upon pathogen recognition by legume plants is an intricate and complicated process that obeys to genotype, plant growth stage, and the kind of pathogen involved. For instance, in an anthracnose-resistant line of *P. vulgaris*, Spm synthase, and *SAMDC* were downregulated in the first stages of infection by the hemi-biotrophic fungus *Colletotrichum lindemuthianum*, compared to a susceptible line (Padder et al., 2016). However, in later stages *ADC, ODC*, and Spd/Spm synthase transcripts were up-regulated, while those encoding hydroxycinnamoyl transferases and N-acetyl transferases (enzymes involved in conjugation of PAs to hydroxycinnamic acid and acetyl groups, respectively) were down-regulated, indicating that PAs conjugation has no relation to plant resistance. Nevertheless, PAs metabolism is not always directly linked to plant resistance. In this sense, in tolerant and susceptible lines of *L. japonicus* and *M. truncatula* confronted with the bacteria *Pseudomonas syringae*, PAs synthesis, and degradation were equally up-regulated (Bordenave et al., 2013; Nemchinov et al., 2017). These discrepancies challenge the understanding of the real contribution of PAs to plant resistance. For instance, Spd and Spm synthase activities were also up-regulated in soybean against the cyst nematode *Heterodera glycines* (Wan et al., 2015); *SAMDC* and *ADC* transcripts were down-regulated, and the *PAO* gene family was up-regulated in response to the Asian soybean rust *Phakopsora pachyrhizi* Sydow (Panthee et al., 2009). In turn, *ODC* and Spm synthase down-regulation was reported in *L. sativus* in response to *Ascochyta lathyri* (Almeida et al., 2015) and the *M. truncatula–Phymatotrichopsis omnivore* interaction (Uppalapati et al., 2009), respectively. Thus, further research is required for a deeper understanding of the connection between the regulation of PAs homeostasis and plant biotic stress tolerance.

It is known that defense mechanisms deployed by plants against pathogens depend on the coordinated activation of signaling pathways involving the production of hormones such as SA, JA, and Et. Besides, some reports have demonstrated a clear connection between PAs and hormone metabolism. For instance, Ozawa et al. (2009) demonstrated that the treatment of lima bean (*P. lunatus*) with PAs (particularly Spm), led to an increment on JA levels, which in turn promote the production of volatile terpenoids capable of protecting plants against herbivores. Moreover, co-treatment with Spm and JA led to a higher terpenoids production, which has a high potential as a strategy for herbivores control. JA is also produced because of tissue damage and the attack of pathogenic fungi. Chickpea plants treated with JA provoked a remarkable induction in *DAO* expression and conversely, antagonists of JA (such as SA and ABA) repressed the expression of this gene (Rea et al., 2002). In turn, chickpea plants treated with inhibitors of the Spd synthesis (such as cycloheximide) showed higher levels of Et, which seems to be a consequence of the accelerated SAM production and induction of enzymes participating in Et biosynthesis (Gallardo et al., 1994; Gallardo et al., 1995). These data demonstrate a bidirectional relationship between PAs and defense signaling pathways mediated by hormones.

#### Root Symbiosis

##### Interactions With Arbuscular Mycorrhizal Fungi

Most lineages of terrestrial plants form symbiotic associations with fungi called arbuscular mycorrhizae (AM) belonging to the *phylum Glomeromycota* (Bonfante and Genre, 2008; Wang et al., 2010). AM fungal root colonization requires the mutual recognition of both organisms involved in the symbiosis (Gadkar et al., 2001; Vierheilig and Piche, 2002), the penetration of the root, and the invasion of the cortical cells to form arbuscules: highly branched fungal structures that facilitate the exchange of nutrients between symbionts (Gutjahr and Parniske, 2013).

Several works using legumes have provided evidences indicating that PAs directly stimulate root colonization by AM fungi. This stimulation would occur through at least two different mechanisms: 1) stimulating mycelial growth and adhesion, and 2) inhibiting ethylene (Et) production in roots. Regarding the first mechanism, it was shown that exogenously supplied PAs to the growth medium of *P. sativum* increased the root colonization with *Glomus intraradices* (El-Ghachtouli et al., 1995). Complementarily, the application of DFMO inhibited colonization by *Glomus mosseae* in *P. sativum* roots, and the inhibition was reverted by simultaneous application of Put (El-Ghachtouli et al., 1996). Also, a positive correlation was found between polyamine chain length and their stimulation of fungal development (El-Ghachtouli et al., 1995). Another approach was used in *G. max* roots, where PAs increased in AM-roots. In these roots, silencing arginine decarboxylase gene (*GmADC*) had a negative effect on mycorrhizal colonization, also affecting the normal development of the plant (Salloum et al., 2018). Interestingly, the silencing of *GmDAO* in the same experimental system, promoted arbuscule formation (Salloum et al., 2018). All these studies successfully demonstrated the importance of PAs in the stimulation of mycorrhizal colonization. Additionally, PAs might directly interact with pectinases of the fungi, increasing adhesion or penetration to the plant cell wall, as observed in other plant-fungi interactions (Charnay et al., 1992; Nogales et al., 2008). Exogenous Et applied to both roots growth medium or leaves, have negatively affected AM infection in *M. sativa* (Azcon-Aguilar et al., 1981). Since Spm and Spd has been proved to block Et synthesis in apple fruits (Apelbaum et al., 1981; Mattoo et al., 2018), it is possible that part of the PAs effect could be mediated by the reduction of Et levels in root tissues. The hypothesis about Et inhibiting root colonization has been tested in brz (E107) *P. sativum* mutants, where higher Et levels were correlated with lower mycorrhizal colonization (Foo et al., 2016; Morales Vela et al., 2007). Interestingly, in *ein2* (Et insensitive) mutant plants treated with Ethephon, mycorrhizal colonization was not reduced as in the wild type, although PAs were not study in these mutants. In consequence, the role of PAs reducing root colonization by Et has been barely suggested. Further research needs to be done to confirm if Et is required to the role of PAs in AM-*ein2* pea mutants treated or not with Ethephon.

As previously discuss, PAs are involved in stress tolerances responses, and in the case of mycorrhizal interaction, PAs could be mediating mitigation effects. A few works have addressed the putative role of PAs on the AM-induced mitigation of plant abiotic stress in legumes. In mycorrhizal *Trigonella foenum-graecum*, a reduction of salt-induced damage of the cell membrane ultrastructure was attributed to a higher PAs (and osmolyte) concentration (Evelin et al., 2013). The inoculation of *V. faba* with selected AM fungi *Funneliformis mosseae* (syn. *Glomus mosseae*), *Rhizophagus intraradices* (syn. *Glomus intraradices*), and *Claroideoglomus etunicatum* (syn. *G. etunicatum*) caused amelioration of the negative effects produced by NaCl (Abeer et al., 2014). In the last work, AM fungi stimulate increases in PAs at a higher extent to that induced by NaCl itself. The higher PAs increase was interpreted by authors as a proof of the protective role of these phytoconstituents against salt stress. Likewise, in *L. tenuis* plants mycorrhized with *Glomus intraradices* under salt stress, a higher content of total free PAs, compared to non-AM ones was reported by Sannazzaro et al. (2007). Since PAs have been proposed as candidates for the regulation of root development under saline situations, authors suggested that the better shape to cope with salt stress displayed by AM *L. tenuis* plants was related to the higher polyamine levels registered.

AM colonization may also increase tolerance of legumes to heavy metals. Attenuation of Pb toxicity in AMF-associated *Calopogonium mucunoides* was associated to a change in amino acids composition favoring metabolic pathways not related to protein, but to PAs biosynthesis (Souza et al., 2014).

As it was pointed out in previous sections PAs play a role in abiotic stress tolerance by regulating water status, ion homeostasis, photosynthesis, and redox status in plant tissues. However, the specific mechanisms linking PAs to abiotic stress mitigation in legumes by AM fungi are not well understood. In this sense, transcriptomic and metabolomic analysis in plants with silenced *ADC* and *DAO*, interacting with AM fungi under abiotic stress could be a good starting point for new research hypothesis. Importantly, in contrast to *Arabidopsis* and other plant families, legumes will allow the study of PAs roles in the triple interaction with rhizobia and AM fungi.

##### Interaction With Rhizobia

Legumes may establish symbiotic associations with 98 species of NFB (Weir, 2011). A new plant organ, the symbiotic root nodule, (Brewin, 1991; Hadri et al., 1998), which hosts bacteria in an optimized environment for fixing atmospheric dinitrogen is formed during the interaction (Jones et al., 2007; Wagner, 2012). One of the first evidences that bacterial PAs could be involved in the nodulation ability of the plant was provided by Ferraioli et al. (2001). These authors studied the response of *P. vulgaris* roots to the inoculation with an *N-acetyl-gamma-glutamyl phosphate reductase* mutant strain of *Rhizobium etli* (unable to grow with ammonium as the sole nitrogen source), and revealed that the early root responses to rhizobial infection were absent with the arginine auxotrophous, but present with the wild-type parent. PAs regulate nodule metabolism mainly through their action on plasma membrane proteins. In soybean nodules, the addition of 200 µM Spd and Put inhibited by 37 and 54% the H^+^-ATPase activity, and both inward and outward ammonium channels, showing that high PAs levels have potential to reduce nitrogen supply to the plant *in vivo* (Whitehead et al., 2001). In addition, the *M. truncatula*-*Sinorhizobium meliloti* symbiotic system provided evidence that the H_2_O_2_ produced by PAs catabolism plays a role in the inhibition of the symbiosis establishment (Hidalgo-Castellanos et al., 2019).

Previous information regarding the role of PAs during early infection stages, their effect (as well as that of GABA) on the regulation of nodule development and efficiency for nitrogen fixation, as well as the expression of genes related with PAs metabolism during the interaction was reviewed by Jiménez-Bremont et al. (2014). That information was centered on *L. japonicus, Galega orientalis, M. sativa*, and *M. truncatula*. More recently, Becerra-Rivera and Dunn (2019) have thoroughly reviewed the types and levels of PAs contents in nodules, their biosynthetic pathways, and the influence of polyamine on traits that are important for the bacterial-host interaction, such as growth capacity, abiotic stress resistance, motility, EPS production, and biofilm formation, addressing some possible intervening mechanisms. These authors also described current knowledge on polyamine synthesis and regulation in rhizobia.

Some works regarding the relationships among the symbiosis with rhizobia, abiotic stresses, and PAs levels were left aside by the mentioned reviews. For example, salinity induced increased PAs levels in *M. sativa/R. meliloti* (Goicoechea et al., 1998) and *L. tenuis/Mesorhizobium tianshanense* (Echeverria et al., 2013). Also, *A. hypogaea* inoculated with *Bradyrhizobium* sp. SEMIA6144 presented a higher (Spd + Spm)/Put relationship in leaves, concomitantly with ameliorated drought symptoms, compared with non-inoculated ones, suggesting that this condition favored tolerance to water deficit (Cesari et al., 2019). In fact, the increase in the relationship (Spd + Spm)/Put has been used as an indicator of plant tolerance to abiotic stresses on other species (Zapata et al., 2004). However, the mechanism involved in the PAs-mediated improvement of tolerance in symbiotic plants is not well understood yet. In this regard, some clues could be obtained by focusing future research on the mutual relationships among salt-induced changes of PAs, and metabolites intervening in carbon and nitrogen metabolisms in nodules, and the entire plant. Glutamate occupies a central position in amino acid plant metabolism, and it is the precursor of arginine, ornithine (both PAs precursors), and proline (**Figure 1**). In turn, arginine (along with asparagine) is a key storage compound in higher plants (Forde and Lea, 2007). Moreover, the group of reactions from glutamate to proline, ornithine, arginine, PAs, and GABA constitutes major pathways for carbon and nitrogen assimilation and partitioning (Rawsthornea et al, 1980; Naliwajski and Skłdowska, 2018). On other hand, there is strong evidence that the stress affects the activity of the enzymes involved in the glutamate metabolism (Forde and Lea, 2007). Therefore, in order to unravel the relationship among rhizobial inoculation, higher PAs levels, and plant tolerance to stress, it would be helpful to compare the regulation of carbon flow in nitrogen metabolism pathways associated with salt-induced alteration of PAs levels, between nodulated and control plants.

### Integrative Tools At the Spotlight: Multiomics Approaches to Decrypt the Interface Legumes—Polyamines-Abiotic and Biotic Stress Interactions

Omics such as transcriptomics and metabolomics have had essential roles in identifying how plant-microbe associations could deviate from classical outcomes in specific conditions (Romero et al., 2017; Rosenberg and Zilber-Rosenberg, 2018). Based on these high-throughput sequencing technologies, an emerging role of PAs in transcriptional regulation and translational modulation of the stress response has been proposed (discussed in Tiburcio et al., 2014). The use of omics is essential to address key questions, such as the specific role of PAs in signaling during abiotic stress (Pál et al., 2015). Transcriptomic assessment of gene expression, perhaps the most prevalent approach to investigate the specific effects of plant coping with stress or development, should not only be restricted to mRNA. This technique has been extensively used to assess the global transcriptional response of PAs transgenic over-expressers during abiotic stress (reviewed in Marco et al., 2011). Nevertheless, other RNA species, such as small RNAs have been reported to play important roles in legume symbiosis, nitrogen fixation, and general plant development. In addition, microRNAs could have specific effects associated with modulation of transcription factors *via* translational arrest. In this scenario, mRNA transcriptomic would fail to identify the biological effects of miRNAs (Hussain et al., 2018).

The development of gene networks based on transcriptomics data has scaled reductionist approaches associated with target sequencing traditional practices. Gene networks based predictions have successfully been applied to survey the uptake, translocation, remobilization, and general regulation of N metabolism in model and crop species (Fukushima and Kusano, 2014). Predictions from transcriptomic data can be weighed with additional complementing technologies. For instance, in soybean, differentially regulated proteins were identified by integrated proteomics and metabolomics during hormone treatment presenting a supported model of altered flavonoid and isoflavonoid metabolism upon Et and ABA treatment (Gupta et al., 2018). Another interesting example of complementation of transcriptomics and metabolomics data in legumes was reported during phosphate deficiency in different plant organs (reviewed in Abdelrahman et al., 2018). It is interesting to point out that several of the databases used to assess legume data are restricted to model species, and there is a need to extend these resources to new crops, which are valuable tools to evaluate in-depth omics data (Bagati et al., 2018). In this context, the envisioning of legume molecular targets to be used in biotechnological applications would be more realistic, and the role of PAs probably more deeply understood. Finally, we call to revisit a long-standing proposal, a renewal of the PAs research landscape based on holistic approaches such as system biology (Montanez et al., 2007). Holistic system biology approaches will pave the way to the understanding of the gene-to-metabolite networks that define legume and PAs metabolisms interrelationships.

## Author Contributions

PS and FE wrote the relationships of polyamines with water stress; SM and AR with saline stress; and AnG, FMR, FRR, and LS with biotic stress. The interrelationships between symbionts and polyamines were written by MM, MS, AM, VM, and MC. AyG and CB are the authors of the revision of the roles of polyamines in plants in extreme environments. The design and general coordination of the review work was carried out by AM, PC, JUD, and OR.

## Conflict of Interest

The authors declare that the research was conducted in the absence of any commercial or financial relationships that could be construed as a potential conflict of interest.

## References

[B1] Abdelrahman M. Jogaiah S. Burritt D. J. Tran L. P. (2018). Legume genetic resources and transcriptome dynamics under abiotic stress conditions. Plant Cell Environ. 41, 1972–1983. 10.1111/pce.13123 29314055

[B2] Abeer H. Allah E. F. Alqarawi A. A. El-Didamony G. Alwhibi M. (2014). Alleviation of adverse impact of salinity on faba bean (Vicia faba L.) by arbuscular mycorrhizal fungi. Pak J. Bot. 46, 2003–2013.

[B3] Alcázar R. Altabella T. Marco F. Bortolotti C. Reymond M. Koncz C. (2010). Polyamines: molecules with regulatory functions in plant abiotic stress tolerance. Planta 231, 1237–1249. 10.1007/s00425-010-1130-0 20221631

[B4] Allen G. J. Sanders D. (1996). Control of ionic currents in guard cell vacuoles by cytosolic and luminal calcium. Plant J. 10, 6, 1055–1069, 10. 10.1046/j.1365-313X.1996.10061055.x 9011087

[B5] Almeida N. F. Krezdorn N. Rotter B. Winter P. Rubiales D. Vaz Patto M. C. (2015). Lathyrus sativus transcriptome resistance response to Ascochyta lathyri investigated by deepSuperSAGE analysis. Front. Plant Sci. 6, 178. 10.3389/fpls.2015.00178 25852725 PMC4367168

[B6] An Z. Jing W. Liu Y. Zhang W. (2008). Hydrogen peroxide generated by copper amine oxidase is involved in abscisic acid-induced stomatal closure in Vicia faba. J. Exp. Bot. 59, 815–825. 10.1093/jxb/erm370 18272918

[B7] Apelbaum A. Burgoon A. C. Anderson J. D. Lieberman M. Ben-Arie R. Mattoo A. K. (1981). Polyamines inhibit biosynthesis of ethylene in higher plant tissue and fruit protoplasts. Plant Physiol. 68, 453–456. 10.1104/pp.68.2.453 16661935 PMC427509

[B8] Azcon-Aguilar C. Rodriguez-Navarro D. N. Barea J. M. (1981). Effects of ethrel on the formation and responses to VA mycorrhiza in Medicago and Triticum. Plant Soil 60, 461–468. 10.1007/BF02149642

[B9] Bagati S. Mahajan R. Nazir M. Dar A. A. Zargar S. M., (2018). “'Omics': a gateway towards abiotic stress tolerance,” in in Abiotic stress-mediated sensing and signaling in plants: an omics perspective (Singapore: Springer), 1–45. 10.1007/978-981-10-7479-0_1

[B10] Barker D. G. Bianchi S. Blondon F. Dattée Y. Duc G. Essad S. (1990). Medicago truncatula, a model plant for studying the molecular genetics of the Rhizobium-legume symbiosis. Plant Mol. Biol. Rep 8, 40–49. 10.1007/BF02668879

[B11] Basra R. K. Basra A. S. Malik C. P. Grover I. S. (1997). Are polyamines involved in the heat-shock protection of mung bean seedlings? *Bot*. Bull. Acad. Sin. 38, 165–169.

[B12] Becerra-Rivera V. A. Dunn M. F. (2019). Polyamine biosynthesis and biological roles in rhizobia. FEMS Microbiol. Lett. 366, p.fnz084. 10.1093/femsle/fnz084 31062028

[B13] Benzarti S. Mohri S. Ono Y. (2008). Plant response to heavy metal toxicity: comparative study between the hyperaccumulator Thlaspi caerulescens (ecotype Ganges) and non-accumulator plants: lettuce, radish, and alfalfa. Environ. Toxicol. Int. J. 23, 607–616. 10.1002/tox.20405 18528911

[B14] Bitrián M. Zarza X. Altabella T. Tiburcio A. F. Alcázar R. (2012). Polyamines under abiotic stress: metabolic crossroads and hormonal crosstalks in plants. Metabolites 2, 516–528. 10.3390/metabo2030516 24957645 PMC3901213

[B15] Bonfante P. Genre A. (2008). Plants and arbuscular mycorrhizal fungi: an evolutionary-developmental perspective. Trends Plant Sci. 13, 492–498. 10.1016/j.tplants.2008.07.001 18701339

[B16] Bordenave C. D. Escaray F. J. Menendez A. B. Serna E. Carrasco P. Ruiz O. A. (2013). Defense Responses in Two Ecotypes of Lotus japonicus against Non-Pathogenic Pseudomonas syringae. PloS One 8, p.e83199. 10.1371/journal.pone.0083199 PMC3859661 24349460

[B17] Bouche N. Fromm H. (2004). GABA in plants: just a metabolite? Trends Plant Sci. 9, 110–115. 10.1016/j.tplants.2004.01.006 15003233

[B18] Brewin N. J. (1991). Development of the legume root nodule. Annu. Rev. Cell Biol. 7, 191–226. 10.1146/annurev.cb.07.110191.001203 1809347

[B19] Calzadilla P. I. Gazquez A. Maiale S. J. Ruiz O. A. Bernardina M. A., (2014). “Polyamines as indicators and modulators of the abiotic stress in plants,” in Plant adapt. to environ. chang. significance amin. acids their deriv. CABI (Wallingford, UK: CABI-Centre for Agricultural Bioscience International), 109–128. 10.1079/9781780642734.0109

[B20] Campestre M. P. Bordenave C. D. Origone A. C. Menéndez A. B. Ruiz O. A. Rodríguez A. A. (2011). Polyamine catabolism is involved in response to salt stress in soybean hypocotyls. J. Plant Physiol. 168, 1234–1240. 10.1016/j.jplph.2011.01.007 21324548

[B21] Cesari A. B. Paulucci N. S. López-Gómez M. Hidalgo-Castellanos J. Plá C. L. Dardanelli M. S. (2019). Performance of Bradyrhizobium and Bradyrhizobium-Azospirillum in alleviating the effects of water-restrictive conditions during the early stages of Arachis hypogaea growth. J. Plant Growth Regul. 38, 1–13. 10.1007/s00344-019-09939-4

[B22] Charnay D. Nari J. Noat G. (1992). Regulation of plant cell-wall pectin methyl esterase by polyamines–interactions with the effects of metal ions. Eur. J. Biochem. 205, 711–714. 10.1111/j.1432-1033.1992.tb16833.x 1572369

[B23] Chen D. Shao Q. Yin L. Younis A. Zheng B. (2019a). Polyamine function in plants: metabolism, regulation on development, and roles in abiotic stress responses. Front. Plant Sci. 9, 1945. 10.3389/fpls.2018.01945 30687350 PMC6335389

[B24] Chen J. Li H. Yang K. Wang Y. Yang L. Hu L. (2019b). Melatonin facilitates lateral root development by coordinating PAO-derived hydrogen peroxide and Rboh-derived superoxide radical. Free Radical Bio. Med. 143, 534–544. 10.1016/j.freeradbiomed.2019.09.011 31520769

[B25] Chmielowska-Bąk J. Lefèvre I. Lutts S. Deckert J. (2013). Short term signaling responses in roots of young soybean seedlings exposed to cadmium stress. J. Plant Physiol. 170, 1585–1594. 10.1016/j.jplph.2013.06.019 23942356

[B26] Choudhary A. Singh R. P. (2000). Cadmium-induced changes in diamine oxidase activity and polyamine levels in Vigna radiata Wilczek seedlings. J. Plant Physiol. 156, 704–710. 10.1016/S0176-1617(00)80235-7

[B27] Cohen S. S. (1998). Guide to the Polyamines. Oxford: Oxford University Press.

[B28] Couée I. Hummel I. Sulmon C. Gouesbet G. El Amrani A. (2004). Involvement of polyamines in root development. Plant Cell Tiss. Org. Cult. 76, 1–10. 10.1023/A:1025895731017

[B29] da Silva J. Saccini V. A. V. dos Santos D. M. M. (2015). Temperature stress in accumulation of free proline of pigeonpea seedlings from seeds treated with polyamines. Semin Ciências Agrárias 36, 103–122. 10.5433/1679-0359.2015v36n1p103

[B30] Dawood M. G. El-Awadi M. E. (2015). Alleviation of salinity stress on Vicia faba L. Plants Via Seed Priming Melatonin Acta Biol. Colomb. 20, 223–235. 10.15446/abc.v20n2.43291

[B31] Dias D. A. Hill C. B. Jayasinghe N. S. Atieno J. Sutton T. Roessner U. (2015). Quantitative profiling of polar primary metabolites of two chickpea cultivars with contrasting responses to salinity. J. Chromatogr. B 1000, 1–13. 10.1016/j.jchromb.2015.07.002 26204234

[B32] Duque A. S. López-Gómez M. Kráčmarová J. Gomes C. N. Araújo S. S. Lluch C. (2016). Genetic engineering of polyamine metabolism changes Medicago truncatula responses to water deficit. Plant Cell Tissue Organ Cult. 127, 681–690. 10.1007/s11240-016-1107-1

[B33] Duranti M. Gius C. (1997). Legume seeds: protein content and nutritional value. F Crop Res. 53, 31–45. 10.1016/S0378-4290(97)00021-X

[B34] Echeverria M. Sannazzaro A. I. Ruiz O. A. Menéndez A. B. (2013). Modulatory effects of mesorhizobium tianshanense and glomus intraradices on plant proline and polyamine levels during early plant response of Lotus tenuis to salinity. Plant Soil 364, 69–79. 10.1007/s11104-012-1312-6

[B35] El-Ghachtouli N. Paynot M. Morandi D. Martin-Tanguy J. Gianinazzi S. (1995). The effect of polyamines on endomycorrhizal infection of wild-type Pisum sativum, cv. Frisson (nod+ myc+) and two mutants (nod– myc+ and nod– myc–). Mycorrhiza 5, 189–192. 10.1007/s005720050058

[B36] El-Ghachtouli N. Martin-Tanguy J. Paynot M. Gianinazzi S. (1996). First report of the inhibition of arbuscular mycorrhizal infection of Pisum sativum by specific and irreversible inhibition of polyamine biosynthesis or by gibberellic acid treatment. FEBS Lett. 385, 189–192. 10.1016/0014-5793(96)00379-1 8647248

[B37] Escaray F. J. Menendez A. B. Gárriz A. Pieckenstain F. L. Estrella M. J. Castagno L. N. (2012). Ecological and agronomic importance of the plant genus Lotus. Its application in grassland sustainability and the amelioration of constrained and contaminated soils. Plant Sci. 182, 121–133. 10.1016/j.plantsci.2011.03.016 22118623

[B38] Espasandin F. D. Maiale S. J. Calzadilla P. Ruiz O. A. Sansberro P. A. (2014). Transcriptional regulation of 9-cis-epoxycarotenoid dioxygenase (NCED) gene by putrescine accumulation positively modulates ABA synthesis and drought tolerance in Lotus tenuis plants. Plant Physiol. Biochem. 76, 29–35. 10.1016/j.plaphy.2013.12.018 24448322

[B39] Evelin H. Giri B. Kapoor R. (2013). Ultrastructural evidence for AMF mediated salt stress mitigation in Trigonella foenum-graecum. Mycorrhiza 23, 71–86. 10.1007/s00572-012-0449-8 22733451

[B40] FAO (2008), Land and Plant Nutrition Management Service. ProSoil-Problem Soils Database, FAO (http//www.fao.org).

[B41] Farhangi-Abriz S. Faegi-Analou R. Nikpour-Rashidabad N. (2017). Polyamines, affected the nitrogen partioning, protein accumulation and amino acid composition of mung bean under water stress. J. Crop Sci. Biotechnol. 20, 279–285. 10.1007/s12892-017-0079-0

[B42] Fariduddin Q. Varshney P. Yusuf M. Ahmad A. (2013). Polyamines: potent modulators of plant responses to stress. J. Plant Interact. 8, 1–16. 10.1080/17429145.2012.716455

[B43] Ferraioli S. Taté R. Caputo E. Lamberti A. Riccio A. Patriarca E. J. (2001). The rhizobium etli argC gene is essential for arginine biosynthesis and nodulation of phaseolus vulgaris. Mol. Plant-Microbe Interact. 14, 250–254. 10.1094/MPMI.2001.14.2.250 11204789

[B44] Flexas J. Gago J. (2018). A role for ecophysiology in the'omics' era. Plant J. 96, 251–259. 10.1111/tpj.14059 30091802

[B45] Flowers T. J. (2004). Improving crop salt tolerance. J. Exp. Bot. 55, 307–319. 10.1093/jxb/erh003 14718494

[B46] Foo E. McAdam E. L. Weller J. L. Reid J. B. (2016). Interactions between ethylene, gibberellins, and brassinosteroids in the development of rhizobial and mycorrhizal symbioses of pea. J. Exp. Bot. 67, 2413–2424. 10.1093/jxb/erw047 26889005 PMC4809293

[B47] Forde B. G. Lea P. J. (2007). Glutamate in plants: metabolism, regulation, and signalling. J. Exp. Bot. 58, 2339–2358. 10.1093/jxb/erm121 17578865

[B48] Franchin C. Fossati T. Pasquini E. Lingua G. Castiglione S. Torrigiani P. (2007). High concentrations of zinc and copper induce differential polyamine responses in micropropagated white poplar (Populus alba). Physiol. Plant 130, 77–90. 10.1111/j.1399-3054.2007.00886.x

[B49] Fukushima A. Kusano M. (2014). A network perspective on nitrogen metabolism from model to crop plants using integrated "omics" approaches. J. Exp. Bot. 65, 5619–5630. 10.1093/jxb/eru322 25129130

[B50] Gadkar V. David-Schwartz R. Kunik T. Kapulnik Y. (2001). Arbuscular mycorrhizal fungal colonization. Factors involved in host recognition. Plant Physiol. 127, 1493–1499. 10.1104/pp.010783 11743093 PMC1540182

[B51] Gallardo M. Gallardo M. E. Matilla A. J. de Rueda P. M. Sánchez-Calle I. M. (1994). Inhibition of polyamine synthesis by cyclohexylamine stimulates the ethylene pathway and accelerates the germination of Cicer arietinum seeds. Physiol. Plant 91, 9–16. 10.1111/j.1399-3054.1994.tb00652.x

[B52] Gallardo M. de Rueda P. M. Matilla A. J. Sánchez-Calle I. M. (1995). Alterations of the ethylene pathway in germinating thermoinhibited chick-pea seeds caused by the inhibition of polyamine biosynthesis. Plant Sci. 104, 169–175. 10.1016/0168-9452(94)04019-D

[B53] Galston A. W. Sawhney R. K. (1990). Polyamines in plant physiology. Plant Physiol. 94, 406–410. 10.1104/pp.94.2.406 11537482 PMC1077246

[B54] Garcıa-Mata C. Lamattina L. (2002). Nitric oxide and abscisic acid cross talk in guard cells. Plant Physiol. 128, 790–792. 10.1104/pp.011020 11891235 PMC1540215

[B55] Geilfus C.-M. Niehaus K. Gödde V. Hasler M. Zörb C. Gorzolka K. (2015). Fast responses of metabolites in Vicia faba L. to moderate NaCl stress. Plant Physiol. Biochem. 92, 19–29. 10.1016/j.plaphy.2015.04.008 25900421

[B56] Gibson K. E. Kobayashi H. Walker G. C. (2008). Molecular determinants of a symbiotic chronic infection. Annu. Rev. Genet. 42, 413. 10.1146/annurev.genet.42.110807.091427 18983260 PMC2770587

[B57] Gill S. S. Tuteja N. (2010). Reactive oxygen species and antioxidant machinery in abiotic stress tolerance in crop plants. Plant Physiol. Biochem. 48, 909–930. 10.1016/j.plaphy.2010.08.016 20870416

[B58] Goicoechea N. Szalai G. Antolín M. C. Sánchez-Díaz M. Paldi E. (1998). Influence of arbuscular mycorrhizae and Rhizobium on free polyamines and proline levels in water-stressed alfalfa. J. Plant Physiol. 153, 706–711. 10.1016/S0176-1617(98)80224-1

[B59] Gonzalez M. E. Marco F. Minguet E. G. Sorli P. C. Blázquez M. A. Carbonell J. (2011). Perturbation of spermine synthase gene expression and transcript profiling provide new insights on the role of the tetraamine spermine in Arabidopsis thaliana defense against Pseudomonas viridiflava. Plant Physiol. 156, pp–110. 10.1104/pp.110.171413 PMC3149955 21628628

[B60] Graham P. H. Vance C. P. (2003). Legumes: importance and constraints to greater use. Plant Physiol. 131, 872–877. 10.1104/pp.017004 12644639 PMC1540286

[B61] Granero S. Domingo J. L. (2002). Levels of metals in soils of Alcalá de Henares, Spain: Human health risks. Environ. Int. 28, 159–164. 10.1016/S0160-4120(02)00024-7 12222612

[B62] Groppa M. D. Tomaro M. L. Benavides M. P. (2007). Polyamines and heavy metal stress: the antioxidant behavior of spermine in cadmium-and copper-treated wheat leaves. Biometals 20, 185–195. 10.1007/s10534-006-9026-y 17068660

[B63] Guo Z. F. Tan J. L. Zhuo C. L. Wang C. Y. Xiang B. Wang Z. Y. (2014). Abscisic acid, H2O2 and nitric oxide interactions mediated cold-induced S-adenosylmethionine synthetase in Medicago sativa subsp. falcata that confers cold tolerance through up-regulating polyamine oxidation. Plant Biotechnol. J. 12, 601–612. 10.1111/pbi.12166 24517136

[B64] Gupta R. Min C. W. Kramer K. Agrawal G. K. Rakwal R. Park K. (2018). A multi-omics analysis of glycine max leaves reveals alteration in flavonoid and isoflavonoid metabolism upon ethylene and abscisic acid treatment. Proteomics 18, 1700366. 10.1002/pmic.201700366 29457974

[B65] Gutjahr C. Parniske M. (2013). Cell and developmental biology of arbuscular mycorrhiza symbiosis. Annu. Rev. Cell Dev. Biol. 29, 593–617. 10.1146/annurev-cellbio-101512-122413 24099088

[B66] Hadri A.-E. Spaink H. P. Bisseling T. Brewin N. J., (1998). “Diversity of root nodulation and rhizobial infection processes,” in The Rhizobiaceae (Dordrecht: Springer), 347–360. 10.1007/978-94-011-5060-6_18

[B67] Hamdani S. Yaakoubi H. Carpentier R. (2011). Polyamines interaction with thylakoid proteins during stress. J. Photochem. Photobiol. B Biol. 104, 314–319. 10.1016/j.jphotobiol.2011.02.007 21377374

[B68] Handberg K. Stougaard J. (1992). Lotus japonicus, an autogamous, diploid legume species for classical and molecular genetics. Plant J. 2, 487–496. 10.1111/j.1365-313X.1992.00487.x

[B69] Hayat S. Hayat Q. Alyemeni M. N. Wani A. S. Pichtel J. Ahmad A. (2012). Role of proline under changing environments: a review. Plant Signal. Behav. 7, 1456–1466. 10.4161/psb.21949 22951402 PMC3548871

[B70] Hidalgo-Castellanos J. Marín-Peña A. Jiménez-Jiménez S. Herrera-Cervera J. A. López-Gómez M. (2019). Polyamines oxidation is required in the symbiotic interaction Medicago truncatula-Sinorhizobium meliloti but does not participate in the regulation of polyamines level under salinity. Plant Growth Regul. 88, 1–11. 10.1007/s10725-019-00508-z

[B71] Hoekenga O. A. Vision T. J. Shaff J. E. Monforte A. J. Lee G. P. Howell S. H. (2003). Identification and characterization of aluminum tolerance loci in Arabidopsis (Landsberg erecta× Columbia) by quantitative trait locus mapping. A Physiologically Simple But Genetically Complex Trait Plant Physiol. 132, 936–948. 10.1104/pp.103.023085 12805622 PMC167032

[B72] Hossain M. A. Piyatida P. da Silva J. A. T. Fujita M. (2012). Molecular mechanism of heavy metal toxicity and tolerance in plants: central role of glutathione in detoxification of reactive oxygen species and methylglyoxal and in heavy metal chelation. J. Bot. 2012. 10.1155/2012/872875

[B73] Hussain S. S. Ali M. Ahmad M. Siddique K. H. M. (2011). Polyamines: natural and engineered abiotic and biotic stress tolerance in plants. Biotechnol. Adv. 29, 300–311. 10.1016/j.biotechadv.2011.01.003 21241790

[B74] Hussain S. S. Hussain M. Irfan M. Siddique K. H. M., (2018). “Legume, microbiome, and regulatory functions of miRNAs in systematic regulation of symbiosis,” in Plant microbiome: stress response (Singapore: Springer), 255–282. 10.1007/978-981-10-5514-0_12

[B75] Jancewicz A. Gibbs N. Masson P. (2016). Cadaverine's functional role in plant development and environmental response. Front. Plant Sci. 7, 870. 10.3389/fpls.2016.00870 27446107 PMC4914950

[B76] Jiao Y. Bai Z. Xu J. Zhao M. Khan Y. Hu Y. (2018). Metabolomics and its physiological regulation process reveal the salt-tolerant mechanism in Glycine soja seedling roots. Plant Physiol. Biochem. 126, 187–196. 10.1016/j.plaphy.2018.03.002 29525442

[B77] Jiménez-Bremont J. F. Ruiz O. A. Rodríguez-Kessler M. (2007). Modulation of spermidine and spermine levels in maize seedlings subjected to long-term salt stress. Plant Physiol. Biochem. 45, 812–821. 10.1016/j.plaphy.2007.08.001 17890098

[B78] Jiménez Bremont J. F. Marina M. Guerrero-González M. de la L. Rossi F. R. Sánchez-Rangel D., (2014). Physiological and molecular implications of plant polyamine metabolism during biotic interactions. Front. Plant Sci. 5, 95. 10.3389/fpls.2014.00095 24672533 PMC3957736

[B79] Jones K. M. Kobayashi H. Davies B. W. Taga M. E. Walker G. C. (2007). How rhizobial symbionts invade plants: the Sinorhizobium-Medicago model. Nat. Rev. Microbiol. 5, 619. 10.1038/nrmicro1705 17632573 PMC2766523

[B80] Khan M. A. Gemenet D. C. Villordon A. (2016). Root system architecture and abiotic stress tolerance: current knowledge in root and tuber crops. Front. Plant Sci. 7, 1584. 10.3389/fpls.2016.01584 27847508 PMC5088196

[B81] Kochian L. V. Hoekenga O. A. Pineros M. A. (2004). How do crop plants tolerate acid soils?Mechanisms of aluminum tolerance and phosphorous efficiency. Annu. Rev. Plant Biol. 55, 459–493. 10.1146/annurev.arplant.55.031903.141655 15377228

[B82] Königshofer H. Lechner S. (2002). Are polyamines involved in the synthesis of heat-shock proteins in cell suspension cultures of tobacco and alfalfa in response to high-temperature stress? Plant Physiol. Biochem. 40, 51–59. 10.1016/S0981-9428(01)01347-X

[B83] Kusano T. Berberich T. Tateda C. Takahashi Y. (2008). Polyamines: essential factors for growth and survival. Planta 228, 367–381. 10.1007/s00425-008-0772-7 18594857

[B84] Legocka J. Sobieszczuk-Nowicka E. (2012). Sorbitol and NaCl stresses affect free, microsome-associated and thylakoid-associated polyamine content in Zea mays and Phaseolus vulgaris. Acta Physiol. Plant 34, 1145–1151. 10.1007/s11738-011-0911-9

[B85] Li Z. Peng Y. Zhang X.-Q. Pan M.-H. Ma X. Huang L.-K. (2014a). Exogenous spermidine improves water stress tolerance of T. repens ('Trifolium repens' L.) involved in antioxidant defence, gene expression and proline metabolism. Plant Omics 7, 517.

[B86] Li Z. Peng Y. Zhang X.-Q. Ma X. Huang L.-K. Yan Y.-H. (2014b). Exogenous spermidine improves seed germination of T. repens under water stress via involvement in starch metabolism, antioxidant defenses and relevant gene expression. Molecules 19, 18003–18024. 10.3390/molecules191118003 25379640 PMC6271027

[B87] Li Z. Jing W. Peng Y. Zhang X. Q. Ma X. Huang L. K. (2015a). Spermine alleviates drought stress in T. repens with different resistance by influencing carbohydrate metabolism and dehydrins synthesis. PloS One 10, e0120708. 10.1371/journal.pone.0120708 25835290 PMC4383584

[B88] Li Z. Zhang Y. Peng D. Wang X. Peng Y. He X. (2015b). Polyamine regulates tolerance to water stress in leaves of white clover associated with antioxidant defense and dehydrin genes via involvement in calcium messenger system and hydrogen peroxide signaling. Front. Physiol. 6, 280. 10.3389/fphys.2015.00280 26528187 PMC4600907

[B89] Li Z. Zhang Y. Zhang X. Peng Y. Merewitz E. Ma X. (2016a). The alterations of endogenous polyamines and phytohormones induced by exogenous application of spermidine regulate antioxidant metabolism, metallothionein and relevant genes conferring drought tolerance in white clover. Environ. Exp. Bot. 124, 22–38. 10.1016/j.envexpbot.2015.12.004

[B90] Li Z. Zhang Y. Xu Y. Zhang X. Peng Y. Ma X. (2016b). Physiological and iTRAQ-based proteomic analyses reveal the function of spermidine on improving drought tolerance in white clover. J. Proteome Res. 15, 1563–1579. 10.1021/acs.jproteome.6b00027 27030016

[B91] Li Z. Li Y. Zhang Y. Cheng B. Peng Y. Zhang X. (2018a). Indole-3-acetic acid modulates phytohormones and polyamines metabolism associated with the tolerance to water stress in white clover. Plant Physiol. Biochem. 129, 251–263. 10.1016/j.plaphy.2018.06.009 29906775

[B92] Li Z. Zhang Y. Peng D. Peng Y. Zhang X. Ma X. (2018b). The inhibition of polyamine biosynthesis weakens the drought tolerance in white clover (Trifolium repens) associated with the alteration of extensive proteins. Protoplasma 255, 803–817. 10.1007/s00709-017-1186-9 29181726

[B93] Li Z. Zhu Y. He X. Yong B. Peng Y. Zhang X. (2019). The hydrogen sulfide, a downstream signaling molecule of hydrogen peroxide and nitric oxide, involves spermidine-regulated transcription factors and antioxidant defense in white clover in response to dehydration. Environ. Exp. Bot. 161, 255–264. 10.1016/j.envexpbot.2018.06.036

[B94] Lin C. C. Kao C. H. (1999). Excess copper induces an accumulation of putrescine in rice leaves. Bot. Bull. Acad. Sin. 40, 213–218.

[B95] Liu K. Fu H. Bei Q. Luan S. (2000). Inward potassium channel in guard cells as a target for polyamine regulation of stomatal movements. Plant Physiol. 124, 1315–1326. 10.1104/pp.124.3.1315 11080307 PMC59229

[B96] Liu C. Zhao L. Yu G. (2011). The dominant glutamic acid metabolic flux to produce γ-amino butyric acid over proline in Nicotiana tabacum leaves under water stress relates to its significant role in antioxidant activity. J. Integr. Plant Biol. 53, 608–618. 10.1111/j.1744-7909.2011.01049.x 21564543

[B97] Liu A. Xiao Z. Li M. Wong F. Yung W. Ku Y. (2019). Transcriptomic reprogramming in soybean seedlings under salt stress. Plant Cell Environ. 42, 98–114. 10.1111/pce.13186 29508916

[B98] Llanes A. Arbona V. Gómez-Cadenas A. Luna V. (2016). Metabolomic profiling of the halophyte Prosopis strombulifera shows sodium salt-specific response. Plant Physiol. Biochem. 108, 145–157. 10.1016/j.plaphy.2016.07.010 27428369

[B99] López-Gómez M. Cobos-Porras L. Hidalgo-Castellanos J. Lluch C. (2014). Occurrence of polyamines in root nodules of Phaseolus vulgaris in symbiosis with Rhizobium tropici in response to salt stress. Phytochemistry 107, 32–41. 10.1016/j.phytochem.2014.08.017 25220497

[B100] López-Gómez M. Cobos-Porras L. Prell J. Lluch C. (2016). Homospermidine synthase contributes to salt tolerance in free-living Rhizobium tropici and in symbiosis with Phaseolus vulgaris. Plant Soil 404, 413–425. 10.1007/s11104-016-2848-7

[B101] LPWG (2017)Phylogeny and classification of the Leguminosae. Taxon 66, 44–77.

[B102] Maiale S. Sánchez D. H. Guirado A. Vidal A. Ruiz O. (2004). Spermine accumulation under salt stress. J. Plant Physiol. 161, 35–42. 10.1078/0176-1617-01167 15002662

[B103] Marco F. Alcazar R. Tiburcio A. F. Carrasco P. (2011). Interactions between polyamines and abiotic stress pathway responses unraveled by transcriptome analysis of polyamine overproducers. Omi J. Integr. Biol. 15, 775–781. 10.1089/omi.2011.0084 PMC3229227 22011340

[B104] Mattoo A. K. White W. B. (2018). Regulation of Ethylene Biosynthesis. Plant Hormone Ethylene. (Florida: CRC Press), 21–42. 10.1201/9781351075763-2

[B105] Minocha R. Majumdar R. Minocha S. C. (2014). Polyamines and abiotic stress in plants: a complex relationship. Front. Plant Sci. 5, 175. 10.3389/fpls.2014.00175 24847338 PMC4017135

[B106] Molina C. (2008). High-throughput genome-wide expression analysis of a non-model organism,” in The chickpea root and nodule transcriptome under salt and drought stress (Doctoral dissertation, Dissertation, 230 pages. Available online: www.ub.unifrankfurt.de/dissertationen). Frankfurt, Germany: Goethe University Frankfurt.

[B107] Montanez R. Sánchez-Jiménez F. Aldana-Montes J. F. Medina M. A. (2007). Polyamines: metabolism to systems biology and beyond. Amino Acids 33, 283–289. 10.1007/s00726-007-0521-4 17514496

[B108] Morales Vela G. Molinero-Rosales N. Ocampo J. A. García Garrido J. M. (2007). Endocellulase activity is associated with arbuscular mycorrhizal spread in pea symbiotic mutants but not with its ethylene content in root. Soil Biol. Biochem. 39, 786–792. 10.1016/j.soilbio.2006.09.028

[B109] Munns R. Tester M. (2008). Mechanisms of salinity tolerance. Annu. Rev. Plant Biol. 59, 651–681. 10.1146/annurev.arplant.59.032607.092911 18444910

[B110] Nahar K. Hasanuzzaman M. Rahman A. Alam M. Mahmud J. A. Suzuki T. (2016). Polyamines confer salt tolerance in mung bean (Vigna radiata L.) by reducing sodium uptake, improving nutrient homeostasis, antioxidant defense, and methylglyoxal detoxification systems. Front. Plant Sci. 7, 1104. 10.3389/fpls.2016.01104 27516763 PMC4964870

[B111] Nahar K. Rahman M. Hasanuzzaman M. Alam M. M. Rahman A. Suzuki T. (2016b). Physiological and biochemical mechanisms of spermine-induced cadmium stress tolerance in mung bean (Vigna radiata L.) seedlings. Environ. Sci. Pollut. Res. 23, 21206–21218. 10.1007/s11356-016-7295-8 27491421

[B112] Nahar K. Hasanuzzaman M. Suzuki T. Fujita M. (2017). Polyamines-induced aluminum tolerance in mung bean: A study on antioxidant defense and methylglyoxal detoxification systems. Ecotoxicology 26, 58–73. 10.1007/s10646-016-1740-9 27819117

[B113] Naliwajski M. R. Skłodowska M. (2018). The relationship between carbon and nitrogen metabolism in cucumber leaves acclimated to salt stress. PeerJ 6, p.e6043. 10.7717/peerj.6043 PMC6292378 30581664

[B114] Nayyar H. Chander S. (2004). Protective effects of polyamines against oxidative stress induced by water and cold stress in chickpea. J. Agron. Crop Sci. 190, 355–365. 10.1111/j.1439-037X.2004.00106.x

[B115] Nayyar H. Satwinder K. Kumar S. Singh K. J. Dhir K. K. (2005). Involvement of polyamines in the contrasting sensitivity of chickpea (Cicer arietinum L.) and soybean (Glycine max (L.) Merrill.) to water deficit stress. Bot. Bull. Acad. Sin 46, 333–338.

[B116] Nemchinov L. G. Shao J. Lee M. N. Postnikova O. A. Samac D. A. (2017). Resistant and susceptible responses in alfalfa (Medicago sativa) to bacterial stem blight caused by Pseudomonas syringae pv. Syringae PloS One 12, e0189781. 10.1371/journal.pone.0189781 29244864 PMC5731681

[B117] Nogales A. Aguirreolea J. Santa María E. Camprubí A. Calvet C. (2008). Response of mycorrhizal grapevine to Armillaria mellea inoculation: disease development and polyamines. Plant Soil 317, 177. 10.1007/s11104-008-9799-6

[B118] Oh D.-H. Dassanayake M. Bohnert H. J. Cheeseman J. M. (2013). Life at the extreme: lessons from the genome. Genome Biol. 13, 241. 10.1186/gb-2012-13-3-241 PMC3439964 22390828

[B119] Ozawa R. Bertea C. M. Foti M. Narayana R. Arimura G.-I. Muroi A. (2009). Exogenous polyamines elicit herbivore-induced volatiles in lima bean leaves: involvement of calcium, H2O2 and Jasmonic acid. Plant Cell Physiol. 50, 2183–2199. 10.1093/pcp/pcp153 19884250

[B120] Padder B. A. Kamfwa K. Awale H. E. Kelly J. D. (2016). Transcriptome profiling of the Phaseolus vulgaris-Colletotrichum lindemuthianum pathosystem. PloS One 11, e0165823. 10.1371/journal.pone.0165823 27829044 PMC5102369

[B121] Pagano A. de Sousa Araújo S. Macovei A. Dondi D. Lazzaroni S. Balestrazzi A. (2019). Metabolic and gene expression hallmarks of seed germination uncovered by sodium butyrate in Medicago truncatula. Plant Cell Env. 42, 259–269. 10.1111/pce.13342 29756644

[B122] Pál M. Szalai G. Janda T. (2015). Speculation: polyamines are important in abiotic stress signaling. Plant Sci. 237, 16–23. 10.1016/j.plantsci.2015.05.003 26089148

[B123] Pál M. Tajti J. Szalai G. Peeva V. Végh B. Janda T. (2018). Interaction of polyamines, abscisic acid and proline under osmotic stress in the leaves of wheat plants. Sci. Rep. 8, 12839. 10.1038/s41598-018-31297-6 30150658 PMC6110863

[B124] Panthee D. R. Marois J. J. Wright D. L. Narváez D. Yuan J. S. Stewart C. N. (2009). Differential expression of genes in soybean in response to the causal agent of Asian soybean rust (Phakopsora pachyrhizi Sydow) is soybean growth stage-specific. Theor. Appl. Genet. 118, 359. 10.1007/s00122-008-0905-1 18853130

[B125] Peiter E. Maathuis F. J. Mills L. N. Knight H. Pelloux J. Hetherington A. M. (2005). The vacuolar Ca 2+-activated channel TPC1 regulates germination and stomatal movement. Nature 434, 404. 10.1038/nature03381 15772667

[B126] Peng D. Wang X. Li Z. Zhang Y. Peng Y. Li Y. (2016). NO is involved in spermidine-induced drought tolerance in white clover via activation of antioxidant enzymes and genes. Protoplasma 253, 1243–1254. 10.1007/s00709-015-0880-8 26338203

[B127] Pinheiro C. Chaves M. M. (2010). Photosynthesis and drought: can we make metabolic connections from available data? J. Exp. Bot. 62, 869–882. 10.1093/jxb/erq340 21172816

[B128] Podlešáková K. Ugena L. Spíchal L. Doležal K. De Diego N. (2019). Phytohormones and polyamines regulate plant stress responses by altering GABA pathway. N Biotechnol. 48, 53–65. 10.1016/j.nbt.2018.07.003 30048769

[B129] Pottosin I. I. Muñiz J. (2002). Higher plant vacuolar ionic transport in the cellular context. Acta Bot. Mex, 37–77. 10.21829/abm60.2002.902

[B130] Pottosin I. Shabala S. (2014). Polyamines control of cation transport across plant membranes: implications for ion homeostasis and abiotic stress signaling. Front. Plant Sci. 5, 154. 10.3389/fpls.2014.00154 24795739 PMC4006063

[B131] Pottosin I. Velarde-Buendía A. M. Bose J. Zepeda-Jazo I. Shabala S. Dobrovinskaya O. (2014a). Cross-talk between reactive oxygen species and polyamines in regulation of ion transport across the plasma membrane: implications for plant adaptive responses. J. Exp. Bot. 65, 1271–1283. 10.1093/jxb/ert423 24465010

[B132] Pottosin I. Velarde-Buendía A. M. Bose J. Fuglsang A. T. Shabala S. (2014b). Polyamines cause plasma membrane depolarization, activate Ca2+-, and modulate H+-ATPase pump activity in pea roots. J. Exp. Bot. 65, 2463–2472. 10.1093/jxb/eru133 24723394

[B133] Pottosin I. Velarde-Buendía A. M. Zepeda-Jazo I. Dobrovinskaya O. Shabala S. (2012). Synergism between polyamines and ROS in the induction of Ca2+ and K+ fluxes in roots. Plant Signal Behav. 7 (60), 1084–1087. 10.4161/psb.21185 22899073 PMC3489633

[B134] Radhakrishnan R. Lee I.-J. (2013). Spermine promotes acclimation to osmotic stress by modifying antioxidant, abscisic acid, and jasmonic acid signals in soybean. J. Plant Growth Regul. 32, 22–30. 10.1007/s00344-012-9274-8

[B135] Radadiya N. Parekh V. B. Dobariya B. Mahatma L. Mahatma M. K. (2016). Abiotic stresses alter expression of S-Adenosylmethionine synthetase gene, polyamines and antioxidant activity in pigeon pea (Cajanus cajan L.). Legum. Res. Int. J. 39, 905–913. 10.18805/lr.v39i6.6640

[B136] Rahman M. Lee S.-H. Ji H. Kabir A. Jones C. Lee K.-W. (2018). Importance of mineral nutrition for mitigating aluminum toxicity in plants on acidic soils: current status and opportunities. Int. J. Mol. Sci. 19, 3073. 10.3390/ijms19103073 PMC6213855 30297682

[B137] Raval S. S. Mahatma M. K. Chakraborty K. Bishi S. K. Singh A. L. Rathod K. J. (2018). Metabolomics of groundnut (Arachis hypogaea L.) genotypes under varying temperature regimes. Plant Growth Regul. 84, 493–505. 10.1007/s10725-017-0356-2

[B138] Rawsthorne S. Minchin F. R. Summerfield R. J. Cookson C. Coombs J. (1980). Carbon and nitrogen metabolism in legume root nodules. Phytochemistry 19, 341–355. 10.1016/0031-9422(80)83181-5

[B139] Rea G. Metoui O. Infantino A. Federico R. Angelini R. (2002). Copper amine oxidase expression in defense responses to wounding and Ascochyta rabiei invasion. Plant Physiol. 128, 865–875. 10.1104/pp.010646 11891243 PMC152200

[B140] Reginato M. A. Abdala G. I. Miersch O. Ruiz O. A. Moschetti E. Luna V. (2012). Changes in the levels of jasmonates and free polyamines induced by Na2SO4 and NaCl in roots and leaves of the halophyte Prosopis strombulifera. Biol. (Bratisl.) 67, 689–697. 10.2478/s11756-012-0052-7

[B141] Rodríguez A. A. Maiale S. J. Menéndez A. B. Ruiz O. A. (2009). Polyamine oxidase activity contributes to sustain maize leaf elongation under saline stress. J. Exp. Bot. 60, 4249–4262. 10.1093/jxb/erp256 19717530

[B142] Romero F. M. Marina M. Pieckenstain F. L. Rossi F. R. Gonzalez M. E. Vignatti P., (2017). “Gaining insight into plant responses to beneficial and pathogenic microorganisms using metabolomic and transcriptomic approaches,” in metabolic engineering for bioactive compounds (Singapore: Springer), 113–140. 10.1007/978-981-10-5511-9_6

[B143] Rosenberg E. Zilber-Rosenberg I. (2018). The hologenome concept of evolution after 10 years. Microbiome 6, 78. 10.1186/s40168-018-0457-9 29695294 PMC5922317

[B144] Rossi F. R. Romero F. M. Ruíz O. A. Marina M. Gárriz A. (2018). “Phenotypic and genotypic characterization of mutant plants in polyamine metabolism genes during pathogenic interactions,” in Polyamines: Methods and Protocols. Eds. Alcázar R. Tiburcio A. F. (New York, NY: Springer New York), 405–416. 10.1007/978-1-4939-7398-9_33 29080183

[B145] Ruttens A. Mench M. Colpaert J. V. Boisson J. Carleer R. Vangronsveld J. (2006). Phytostabilization of a metal contaminated sandy soil. I: Influence of compost and/or inorganic metal immobilizing soil amendments on phytotoxicity and plant availability of metals. Environ. Pollut. 144, 524–532. 10.1016/j.envpol.2006.01.038 16542762

[B146] Salloum M. S. Menduni M. F. Benavides M. P. Larrauri M. Luna C. M. Silvente S. (2018). Polyamines and flavonoids: key compounds in mycorrhizal colonization of improved and unimproved soybean genotypes. Symbiosis 76, 1–11. 10.1007/s13199-018-0558-z

[B147] Sanchez D. H. Cuevas J. C. Chiesa M. A. Ruiz O. A. (2005). Free spermidine and spermine content in Lotus glaber under long-term salt stress. Plant Sci. 168, 541–546. 10.1016/j.plantsci.2004.09.025

[B148] Sanchez D. H. Pieckenstain F. L. Szymanski J. Erban A. Bromke M. Hannah M. A. (2011). Comparative functional genomics of salt stress in related model and cultivated plants identifies and overcomes limitations to translational genomics. PloS One 6, e17094. 10.1371/journal.pone.0017094 21347266 PMC3038935

[B149] Sánchez-Rodríguez E. Romero L. Ruiz J. M. (2016). Accumulation of free polyamines enhances the antioxidant response in fruits of grafted tomato plants under water stress. J. Plant Physiol. 190, 72–78. 10.1016/j.jplph.2015.10.010 26687637

[B150] Sannazzaro A. I. Echeverría M. Albertó E. O. Ruiz O. A. Menéndez A. B. (2007). Modulation of polyamine balance in Lotus glaber by salinity and arbuscular mycorrhiza. Plant Physiol. Biochem. 45, 39–46. 10.1016/j.plaphy.2006.12.008 17303429

[B151] Sato S. Nakamura Y. Kaneko T. Asamizu E. Kato T. Nakao M. (2008). Genome structure of the legume, Lotus japonicus. DNA Res. 15, 227–239. 10.1093/dnares/dsn008 18511435 PMC2575887

[B152] Schmutz J. Cannon S. B. Schlueter J. Ma J. Mitros T. Nelson W. (2010). Genome sequence of the palaeopolyploid soybean. Nature 463, 178. 10.1038/nature08670 20075913

[B153] Shabala S. Cuin T. A. Pottosin I. (2007). Polyamines prevent NaCl-induced K+ efflux from pea mesophyll by blocking non-selective cation channels. FEBS Lett. 581, 1993–1999. 10.1016/j.febslet.2007.04.032 17467698

[B154] Shevyakova N. I. Musatenko L. I. Stetsenko L. A. Vedenicheva N. P. Voitenko L. P. Sytnik K. M. (2013). Effects of abscisic acid on the contents of polyamines and proline in common bean plants under salt stress. Russ J. Plant Physiol. 60, 200–211. 10.1134/S102144371301007X

[B155] Shi S. Q. Shi Z. Jiang Z. P. Qi L. W. Sun X. M. Li C. X. (2010). Effects of exogenous GABA on gene expression of Caragana intermedia roots under NaCl stress: regulatory roles for H2O2 and ethylene production. Plant Cell Environ. 33, 149–162. 10.1111/j.1365-3040.2009.02065.x 19895397

[B156] Shi H. Chan Z. (2014). Improvement of plant abiotic stress tolerance through modulation of the polyamine pathway. J. Integr. Plant Biol. 56, 114–121. 10.1111/jipb.12128 24401132

[B157] Silva J. D. Saccini V. Aparecida V. dos Santos M. Maria D. (2015). Estresse térmico no acúmulo de prolina livre de plântulas de guandu oriundas de sementes tratadas com poliaminas. Semin. Cienc. Agra 36, 103–122. 10.5433/1679-0359.2015v36n1p103

[B158] Singh P. Basu S. Kumar G. (2018). “Polyamines metabolism: a way ahead for abiotic stress tolerance in crop plants,” in biochemical, physiological and molecular avenues for combating abiotic stress tolerance in plants (elsevier) (Cambridge: Academic Press) 39–55. 10.1016/B978-0-12-813066-7.00003-6

[B159] Souza L. A. Camargos L. S. Schiavinato M. A. Andrade S. A. L. (2014). Mycorrhization alters foliar soluble amino acid composition and influences tolerance to Pb in Calopogonium mucunoides. Theor. Exp. Plant Phys. 26, 211–216. 10.1007/s40626-014-0019-x

[B160] Su G. X. Bai X. (2008). Contribution of putrescine degradation to proline accumulation in soybean leaves under salinity. Biol. Plant 52, 796. 10.1007/s10535-008-0156-7

[B161] Tardieu F. Simonneau T. Muller B. (2018). The physiological basis of drought tolerance in crop plants: a scenario-dependent probabilistic approach. Annu. Rev. Plant Biol. 69, 733–759. 10.1146/annurev-arplant-042817-040218 29553801

[B162] Talaat N. B. (2015). Effective microorganisms modify protein and polyamine pools in common bean (Phaseolus vulgaris L.) plants grown under saline conditions. Sci. Hortic. (Amsterdam) 190, 1–10. 10.1016/j.scienta.2015.04.005

[B163] Tiburcio A. F. Altabella T. Bitrián M. Alcázar R. (2014). The roles of polyamines during the lifespan of plants: from development to stress. Planta 240, 1–18. 10.1007/s00425-014-2055-9 24659098

[B164] Torabian S. Shakiba M. R. Nasab A. D. M. Toorchi M. (2018a). Leaf gas exchange and grain yield of common bean exposed to spermidine under water stress. Photosynthetica 56, 1387–1397. 10.1007/s11099-018-0834-4

[B165] Torabian S. Shakiba M. R. Mohammadi Nasab A. D. Toorchi M. (2018b). Exogenous spermidine affected leaf characteristics and growth of common bean under water deficit conditions. Commun. Soil Sci. Plan 49, 1289–1301. 10.1080/00103624.2018.1457157

[B166] Tun N. N. Santa-Catarina C. Begum T. Silveira V. Handro W. Floh E. I. S. (2006). Polyamines induce rapid biosynthesis of nitric oxide (NO) in Arabidopsis thaliana seedlings. Plant Cell Physiol. 47, 346–354. 10.1093/pcp/pci252 16415068

[B167] Ullah A. Manghwar H. Shaban M. Khan A. H. Akbar A. Ali U. (2018). Phytohormones enhanced drought tolerance in plants: a coping strategy. Environ. Sci. Pollut Res. 25, 33103–33118. 10.1007/s11356-018-3364-5 30284160

[B168] Uppalapati S. R. Marek S. M. Lee H.-K. Nakashima J. Tang Y. Sledge M. K. (2009). Global gene expression profiling during Medicago truncatula-Phymatotrichopsis omnivora interaction reveals a role for jasmonic acid, ethylene, and the flavonoid pathway in disease development. Mol. Plant-Microbe Interact. 22, 7–17. 10.1094/MPMI-22-1-0007 19061398

[B169] Vanlerberghe G. C. Martyn G. D. Dahal K. (2016). Alternative oxidase: a respiratory electron transport chain pathway essential for maintaining photosynthetic performance during drought stress. Physiol. Plant 157, 322–337. 10.1111/ppl.12451 27080742

[B170] Varshney R. K. Chen W. Li Y. Bharti A. K. Saxena R. K. Schlueter J. A. (2012). Draft genome sequence of pigeonpea (Cajanus cajan), an orphan legume crop of resource-poor farmers. Nat. Biotechnol. 30, 83. 10.1038/nbt.2022 22057054

[B171] Varshney R. K. Song C. Saxena R. K. Azam S. Yu S. Sharpe A. G. (2013). Draft genome sequence of chickpea (Cicer arietinum) provides a resource for trait improvement. Nat. Biotechnol. 31, 240. 10.1038/nbt.2491 23354103

[B172] Vierheilig H. Piche Y. (2002). “Signalling in arbuscular mycorrhiza: facts and hypotheses,” in Flavonoids in cell function (Boston: Springer), 23–39. 10.1007/978-1-4757-5235-9_3 12083464

[B173] Wagner S. C. (2012). Biological nitrogen fixation. Nat. Educ. Knowl. 3, 15.

[B174] Wan J. Vuong T. Jiao Y. Joshi T. Zhang H. Xu D. (2015). Whole-genome gene expression profiling revealed genes and pathways potentially involved in regulating interactions of soybean with cyst nematode (Heterodera glycines Ichinohe). BMC Genomics 16, 148. 10.1186/s12864-015-1316-8 25880563 PMC4351908

[B175] Wang B. Yeun L. H. Xue J. Liu Y. Ané J. Qiu Y. (2010). Presence of three mycorrhizal genes in the common ancestor of land plants suggests a key role of mycorrhizas in the colonization of land by plants. New Phytol. 186, 514–525. 10.1111/j.1469-8137.2009.03137.x 20059702

[B176] Wei W. Li Q. T. Chu Y. N. Reiter R. J. Yu X. M. Zhu D. H. (2014). Melatonin enhances plant growth and abiotic stress tolerance in soybean plants. J. Exp. Bot. 66, 695–707. 10.1093/jxb/eru392 25297548 PMC4321538

[B177] Weir B. S. (2011). The current taxonomy of rhizobia. New Zealand rhizobia . website http//www.rhizobia.co.nz/taxonomy/rhizobia.html.

[B178] Whitehead L. F. Tyerman S. D. Day D. A. (2001). Polyamines as potential regulators of nutrient exchange across the peribacteroid membrane in soybean root nodules. Funct. Plant Biol. 28, 677–683. 10.1071/PP01025

[B179] Wimalasekera R. Tebartz F. Scherer G. F. E. (2011). Polyamines, polyamine oxidases and nitric oxide in development, abiotic and biotic stresses. Plant Sci. 181, 593–603. 10.1016/j.plantsci.2011.04.002 21893256

[B180] Wolff A. B. Sidirelli M. Paradellis C. Kotzabasis K. (1995). Influence of acid soil on nodule numbers in relation to polyamine and tannin concentrations in roots of Phaseolus vulgaris. Biol. Fertil. Soils 20, 249–252. 10.1007/BF00336085

[B181] Xing S. G. Jun Y. B. Hau Z. W. Liang L. Y. (2007). Higher accumulation of γ-aminobutyric acid induced by salt stress through stimulating the activity of diamine oxidases in Glycine max (L.) Merr. Roots Plant Physiol. Biochem. 45, 560–566. 10.1016/j.plaphy.2007.05.007 17624796

[B182] Yamasaki H. Cohen M. F. (2006). NO signal at the crossroads: polyamine-induced nitric oxide synthesis in plants? Trends Plant Sci. 11, 522–524. 10.1016/j.tplants.2006.09.009 17035070

[B183] Yang R. Guo Q. Gu Z. (2013). GABA shunt and polyamine degradation pathway on γ-aminobutyric acid accumulation in germinating fava bean (Vicia faba L.) under hypoxia. Food Chem. 136, 152–159. 10.1016/j.foodchem.2012.08.008 23017406

[B184] Yang R. Gu Z. Yin Y. (2018). Polyamine degradation pathway regulating growth and GABA accumulation in germinating fava bean under hypoxia-NaCl stress. Jomo Kenyatta University of Agricultural and Technology.

[B185] Yin Y. Yang R. Gu Z. (2014). Calcium regulating growth and GABA metabolism pathways in germinating soybean (Glycine max L.) under NaCl stress. Eur. Food Res. Technol. 239, 149–156. 10.1007/s00217-014-2214-z

[B186] Yong B. Xie H. Li Z. Li Y.-P. Zhang Y. Nie G. (2017). Exogenous Application of GABA Improves PEG-Induced Drought Tolerance Positively Associated with GABA-Shunt, Polyamines, and Proline Metabolism in T. Repens Front. Physiol. 8, 1107. 10.3389/fphys.2017.01107 29312009 PMC5744439

[B187] Young N. D. Debellé F. Oldroyd G. E. D. Geurts R. Cannon S. B. Udvardi M. K. (2011). The Medicago genome provides insight into the evolution of rhizobial symbioses. Nature 480, 520. 10.1038/nature10625 22089132 PMC3272368

[B188] Zafari S. Sharifi M. Chashmi N. A. (2017). Nitric oxide production shifts metabolic pathways toward lignification to alleviate Pb stress in Prosopis farcta. Environ. Exp. Bot. 141, 41–49. 10.1016/j.envexpbot.2017.06.011

[B189] Zapata P. Serrano M. Pretel M. Amoros A. Botella M. (2004). Polyamines and ethylene changes during germination of different plant species under salinity. Plant Sci. 167, 781–788. 10.1016/j.plantsci.2004.05.014

[B190] Zapata P. J. Serrano M. Pretel M. T. Botella M. A. (2008). Changes in free polyamine concentration induced by salt stress in seedlings of different species. Plant Growth Regul. 56, 167–177. 10.1007/s10725-008-9298-z

[B191] Zhang G. W. Xu S. C. Hu Q. Z. Mao W. H. Gong Y. M. (2014). Putrescine plays a positive role in salt-tolerance mechanisms by reducing oxidative damage in roots of vegetable soybean. J. Integr Agric. 13, 349–357. 10.1016/S2095-3119(13)60405-0

[B192] Zhang Y. Li Z. Li Y. P. Zhang X. Q. Ma X. Huang L. K. (2018). Chitosan and spermine enhance drought resistance in white clover, associated with changes in endogenous phytohormones and polyamines, and antioxidant metabolism. Funct. Plant Biol. 45, 1205–1222. 10.1071/FP18012 32291011

[B193] Zhang Q. Liu X. Zhang Z. Liu N. Li D. Hu L. (2019). Melatonin improved waterlogging tolerance in alfalfa (Medicago sativa) by reprogramming polyamine and ethylene metabolism. Front. Plant Sci. 10.3389/fpls.2019.00044 PMC6367245 30774639

[B194] Zeid I. M. Shedeed Z. A. (2006). Response of alfalfa to putrescine treatment under drought stress. Biol. Plant 50, 635. 10.1007/s10535-006-0099-9 19069967

[B195] Zepeda-Jazo I. Velarde-Buendía A. M. Enríquez-Figueroa R. Bose J. Shabala S. Muñiz-Murguía J. (2011). Polyamines interact with hydroxyl radicals in activating Ca2+ and K+ transport across the root epidermal plasma membranes. Plant Phys. 157, 2167–2180. 10.1104/pp.111.179671 PMC3327209 21980172

[B196] Zepeda-Jazo I. Pottosin I. (2018). “Methods related to polyamine control of cation transport across plant membranes,” in Polyamines (New York, NY: Humana Press), 257–276. 10.1007/978-1-4939-7398-9_23 29080173

[B197] Zhang G. W. Xu S. C. Hu Q. Z. Mao W. H. Gong Y. M. (2014). Putrescine plays a positive role in salt-tolerance mechanisms by reducing oxidative damage in roots of vegetable soybean. J. Integr. Agric. 13, 349–357. 10.1016/S2095-3119(13)60405-0

